# Pharmacokinetics and Pharmacodynamics of Nomlabofusp in Non-clinical Studies of Friedreich’s Ataxia

**DOI:** 10.1208/s12248-025-01093-y

**Published:** 2025-06-25

**Authors:** Flavia De Toni, Vanessa Ragaglia, Devin Schecter, Angela S. Miller, Eric Gonzalez, Erik J. Wagner, Xin Xu, R. Mark Payne, Jean-Nicholas Mess, Matthew G. Baile, Adrienne Clements-Egan, Gopi Shankar

**Affiliations:** 1 Larimar Therapeutics Inc., Bala Cynwyd, PA, 3 Bala Plaza East, Suite 506, 19004, United States of America; 2 National Center for Advancing Translational Sciences, National Institutes of Health, Rockville, MD, United States of America; 3 Indiana University School of Medicine, Indianapolis, IN, United States of America; 4 Altasciences Inc., Montreal, QC, CA, Canada

**Keywords:** Animal model, Frataxin, Friedreich’s ataxia, Mitochondria, Non-clinical, Nomlabofusp, Pharmacokinetics

## Abstract

Nomlabofusp is a cell penetrant peptide-based recombinant fusion protein designed to enter cells and deliver human frataxin into the mitochondria of adults and children with Friedreich’s ataxia. In this article we present non-clinical studies evaluating the pharmacology of nomlabofusp, including in a murine striated muscle tissue frataxin knockout model of Friedreich’s ataxia. We demonstrate that subcutaneous administration of nomlabofusp distributes in a dose-dependent manner to several organs including the dorsal root ganglion, heart, and skeletal muscle, which are known to be predominantly affected in Friedreich’s ataxia, as well as to other tissues, including skin. Plasma nomlabofusp concentrations correlated with levels of human frataxin delivered by nomlabofusp into tissues, and the increases in frataxin were correlated amongst tissues, especially with skin. In the knockout mice, we show that the pharmacokinetics and processing of nomlabofusp were comparable with wild type animals and that treatment with nomlabofusp halts the progression of cardiac dysfunction and significantly increased survival. Together, the findings from these non-clinical studies demonstrate that nomlabofusp exposure increases human frataxin in Friedreich’s ataxia-relevant tissues and provide evidence of pharmacologic effects.

## Introduction

Friedreich’s ataxia (FRDA) is a rare, inherited neurological disorder characterized by progressive symptoms including ataxia, scoliosis, diabetes, sensory loss, and hypertrophic cardiomyopathy, the latter being a leading cause of death (1). The disease is caused by mutations in the frataxin gene (*FXN*) with the most common pathogenic variant, the intron-1 GAA repeat expansion, resulting in reduced frataxin protein (FXN) levels in tissues that correlate with symptom onset, severity, and disease progression (2, 3). Therefore, increasing FXN protein levels in these patients represents a potentially promising therapeutic strategy.

Frataxin is nuclear encoded, produced in the cytoplasm, and imported into the mitochondria aided by a mitochondrial targeting sequence (MTS) present at its N-terminus (4, 5). FXN activates enzymes like succinate dehydrogenase (SDH), a mitochondrial specific enzyme complex located on the inner mitochondrial membrane that plays a crucial role in energy production via both the citric acid cycle and electron transport chain (6). Whereas direct interaction with SDH is known (7), it is more likely that FXN plays a broader role as activator of iron-sulfur cluster biogenesis. Iron-sulfur clusters serve as essential cofactors that enable or enhance the activity of many mitochondrial enzymes such as SDH and aconitase (8). Impacted by FXN abundance and activity, iron-sulfur clusters are indispensable for mitochondrial electron transport, enzymatic catalysis, iron regulation, and cellular signaling, underscoring their central role in both mitochondrial and cellular physiology. Thereby FXN affects various cellular processes, including mitochondrial function and reactive oxygen species mitigation. It plays a critical role in early development, as evidenced by the fact that a complete homozygous deletion (knockout, KO) of the *Fxn* gene in animals is embryonic lethal (9). Consequently, several partial or conditional *Fxn-*KO mouse models have been developed but none of these models fully recapitulate the human FRDA phenotype. Nevertheless, they have supported the elucidation of distinct aspects of the disease, such as neuronal or cardiac dysfunction (10, 11). Amongst these is the striated tissue mouse model, produced in *Fxn*^flox/null^::MCK-Cre or *Fxn*^flox/flox^::MCK-Cre transgenic mice (both hereafter referred to as *Fxn-*KO mice), which bears FXN deficits in the heart and skeletal muscle and has been widely researched for its likeness to the cardiac phenotype in patients with FRDA (12, 13). As in patients with FRDA (14), decreased SDH and aconitase activities have been associated with disease progression in this model (12, 15–17).

With the establishment of FXN deficiency as the pathophysiological cause of FRDA (2), it is rational to hypothesize that administration of exogenous functional FXN delivered into the mitochondria could alleviate the deficiency and restore FXN-associated cellular activities, leading to improved clinical outcomes in patients with this disease. Whereas in patients FXN deficiency exists in all tissues, the essential hallmarks of disease progression in FRDA—ataxia and cardiomyopathy—suggest that certain target tissues (heart, skeletal muscle, and neurological tissues such as cerebellum, spinal cord and dorsal root ganglion [DRG]) are more vulnerable to FXN deficiency than others and are therefore considered FRDA-relevant tissues.

Nomlabofusp, a novel recombinant fusion protein (previously referred to as CTI-1601), has been developed as a potential treatment for adults and children with FRDA. It is comprised of a short, trans-activator of transcription (TAT) sequence-based cationic cell penetrant peptide (18) fused through a diglycine linker to the amino-terminus of human FXN (hFXN) containing its native MTS and mature FXN protein. Following administration, nomlabofusp (24.9 kDa) enters cells and is imported into mitochondria where it is fully processed, releasing mature FXN (14.3 kDa). Thus, nomlabofusp was designed to deliver functional FXN to mitochondria in a manner that is indistinguishable from the endogenous mature and active form of FXN. The mechanism of cellular entry and processing of nomlabofusp to mature FXN *in vitro* using cell lines and *in vivo* within buccal tissue of patients with FRDA was recently published (19). Nomlabofusp has also been evaluated in Phase 1 clinical studies in adults with FRDA (20) and in subsequent clinical studies (21) in which nomlabofusp has been demonstrated to raise FXN levels in skin and buccal tissues. However, the relationship between either of these tissues and FRDA-relevant tissues has not been adequately studied. Because it is difficult and impractical to sample FRDA-relevant tissues in humans, we undertook several studies in animals to understand cross-tissue relationships. In this article we present non-clinical studies evaluating the pharmacology of nomlabofusp in the *Fxn-*KO mouse model of FXN deficiency-led cardiomyopathy. Studies were also conducted in wild type (WT) mice, rats and non-human primates using clinically relevant doses to better understand pharmacokinetics (PK), drug distribution, and the resultant effect on FXN levels in various organs.

## Materials and Methods

### Test Article

Nomlabofusp (CTI-1601, CAS No. 2548202–5–5) is a 224 amino acid fusion protein with a molecular formula of C_1098_H_1754_N_322_O_331_S_5_ and molecular weight of 24,922 Da (US Patent 11,459,363).

Nomlabofusp was formulated at 10 mg/mL in vehicle (50 mM sodium acetate, 1% v/v propylene glycol, pH 5.0) or 50 mg/mL in vehicle (21 mM histidine, 259 mM sucrose, 0.05% polysorbate 20, pH 5.8).

### Animals and Experimental Designs

Animal studies were performed according to IACUC approved protocols and in compliance with the Guide for the Care and Use of Laboratory Animals (National Research Council, 1996). Protocol numbers may be available upon request.

#### Nomlabofusp Disposition and Tissue Distribution in Wild Type (WT) or *Fxn*-KO Mice

Male C57BL6 WT adult mice received single nomlabofusp doses of 5 mg/kg via intravenous (*n* = 3/time point), or 10 mg/kg subcutaneous (SC) injection (*n* = 3/time point). *Fxn*-KO mice (Fxn^flox/null^::MCK-Cre [JAX Lab Cat#: 029720]), age ~ 50 days, male and female) received a single nomlabofusp SC dose of 10 mg/kg (*n* = 3/time point) or 50 mg/kg (*n* = 3/time point). The brain, heart, liver and skeletal muscle were harvested 1 h after dosing without prior perfusion.

#### Nomlabofusp Disposition and Tissue Distribution in Rats

Nomlabofusp was administered SC once daily to adult male Sprague Dawley rats at 2 mg/kg (*n* = 17), 5 mg/kg (*n* = 17), or 20 mg/kg (*n* = 17) for 7 days. Untreated rats (*n* = 14) were used as controls. Plasma samples for PK analysis were collected on Study Day 6. On the seventh study day, 2.5 h after the dose, following CO_2_ euthanasia and terminal blood collection, the rats were necropsied for organ collection (cerebellum, cerebrum, DRG, heart, liver, skeletal muscle, and skin). Among all the rats in each dose group, *n* = 6 had the organs harvested without perfusion and *n* = 11 were gravity-perfused with room temperature phosphate buffered saline until circulated/waste perfusion was visually clear from blood. A sub-group of perfused rats (*n* = 5 from each dose level and *n* = 2 rats from the untreated group) had heart and skeletal muscle separated for mitochondrial preparation.

#### Nomlabofusp Disposition and Tissue Distribution in Monkeys

Cynomolgus monkeys (*n* = 6) received nomlabofusp 15 mg/kg SC twice a day. Each monkey served as its own control by receiving vehicle for 2 days (4 doses, Study Days 1 and 2) before receiving nomlabofusp (Study Days 3 through 16). Nomlabofusp SC injections were approximately 12 h apart, except for Day 16, when only 1 dose was administered (in the morning). The site of injection was rotated among 4 separate sites. Plasma, buccal cells, skin, and platelets samples were collected on Study Days 3, 10, and 16 before the AM dose from all monkeys. Cerebrospinal fluid (CSF) was collected from 2 monkeys on Study Day 3 before the first dose of nomlabofusp, and subsequently from 6 monkeys on Study Day 16 before the AM dose.

#### Nomlabofusp Effect on SDH Activity in *Fxn*-KO Mice

Hearts were harvested at necropsy, and mitochondrial extracts (ME) prepared from *Fxn*-KO mice (Fxn^flox/null^::MCK-Cre [JAX Lab Cat #: 029720]) treated starting at 5 weeks of age every 48 h for 14 days by SC administration with nomlabofusp at 10 mg/kg (Group 1, *n* = 1), 2 mg/kg (Group 2, *n* = 7), or 0.4 mg/kg (Group 3, *n* = 8). Another group of *Fxn*-KO mice were treated starting at 6 weeks of age every day for 20 days by SC administration with nomlabofusp, 10 mg/kg (Group 1, *n* = 4), 2 mg/kg (Group 2, *n* = 3), 0.4 mg/kg (Group 3, *n* = 4), or vehicle (Group 4, *n* = 3). Wild type mice (C57BL/6 J mice RRID:IMSR[JAX Lab Cat#: 000664]), serving as a normal control, were untreated (Group 4, *n* = 2). For the skeletal muscle mitochondria experiments, *Fxn*-KO mice starting at 5 weeks of age were treated SC with vehicle (Group 1, *n* = 12), or nomlabofusp, 2 mg/kg (Group 2, *n* = 8), 10 mg/kg (Group 3, *n* = 12), 30 mg/kg (Group 4, *n* = 12), 60 mg/kg (Group 5, *n* = 8), or 100 mg/kg (Group 6, *n* = 8) and WT (C57BL/6 J) mice were treated SC with vehicle (Group 7, *n* = 12) every 2 days for 14 days. SDH activity was measured in heart ME from combined results of samples collected at 1 h and 24 h after the last dose. Skeletal muscle tissues were collected at necropsy, isolated, flash frozen, and stored at −80°C until processing to ME.

#### Nomlabofusp Effect On Cardiac Function of *Fxn*-KO Mice

Starting at 5 weeks of age *Fxn*-KO (Fxn^flox/null^::MCK-Cre [JAX Lab Cat #: 029720]) mice were treated with nomlabofusp 10 mg/kg SC (Group 1, *n* = 8) or vehicle (Group 2, *n* = 8) every 48 h. Wild type mice (C57BL/6 J mice RRID:IMSR_[JAX Lab Cat#: 000664]) were treated with vehicle, (Group 3, *n* = 8) or nomlabofusp, 10 mg/kg SC (Group 4, *n* = 3) every 48 h. All mice were treated for 6 weeks, from 5 to 11 weeks of age. Mice were assessed for cardiac performance by anesthetized echocardiography (*see* Supplementary File 1 *for details*) a week prior to dosing (4 weeks of age) and at 8 weeks of age.

#### Nomlabofusp Effect On Lifespan of *Fxn*-KO Mice

*Fxn*-^flox/flox^::MCK-Cre (*Frda*^L3/L3^::MCK-Cre) KO mice (ablation of the *Fxn* gene was accomplished as previously described by Stram *et al.* (13)) entered the trial at 15 ± 1 days of age and were treated with nomlabofusp 10 mg/kg SC (*n* = 16) or vehicle (*n* = 15) 3 times/week. Mice were dosed until they died or were removed from study by 200 days; they were checked daily and date of death recorded.

### Bioanalysis

#### Nomlabofusp Quantification in Mouse, Rat, and Monkey Plasma

Blood samples were collected into K_2_EDTA tubes, centrifuged, and the plasma collected and kept frozen (≤ −70°C) until analysis. Nomlabofusp was measured in plasma using an Enzyme Linked Immunosorbent Assay (ELISA), an electrochemiluminescence sandwich immunoassay on the Meso Scale Discovery platform, or a hybrid LC–MS/MS assay that included an initial immuno-affinity selection step with an anti-TAT antibody, followed by a trypsin digestion of nomlabofusp to produce the signature peptide SGT, which was quantified by LC–MS/MS (*see* Supplementary File 2 *for details*).

#### FXN Quantification in Mouse, Rat, and Monkey Tissues, Mito-chondrial Extracts, Platelets, and Cerebrospinal Fluid

Whole brain, cerebrum, cerebellum, DRGs, heart, liver tissues, skeletal muscle, and/or skin tissues from mice and rats collected upon necropsy were homogenized in radioimmunoprecipitation assay (RIPA) buffer. Mouse liver mitochondria were isolated using the method of Frezza *et al.* (22) and the pellet was homogenized in RIPA buffer. Rat heart and skeletal muscle ME were prepared using the commercial kit from Abcam (ab110168). Monkey platelets, CSF, skin, and buccal samples were collected at pre-determined time points under anesthesia. Monkey skin tissues were prepared by homogenizing the punch biopsies and buccal cells were prepared by extracting the buccal samples with RIPA buffer. Samples were stored frozen (≤−70°C) until analysis. FXN (human and endogenous) was measured in these tissue samples using a hybrid LC–MS/MS assay that has an initial immuno-affinity selection step using an anti-FXN antibody, followed by trypsin digestion to produce SGT, LGG, and/or GGM peptides. CSF was analyzed without processing for nomlabofusp and FXN using 2 ELISA methods (*see* Supplementary File 2 for details).

### Mouse Mitochondrial SDH Activity

ME were prepared using differential centrifugation with commercially available buffers or prepared according to previously published methods (23, 24). The activity was determined from skeletal and heart ME using an assay kit using modified manufacturer’s (BioVision Inc., Milpitas, CA) protocol (*see* Supplementary File 3 *for details*).

### Immunoprecipitation and Western Blot Analysis

Mitochondrial fractions were resuspended in RIPA buffer with 1 × HALT^™^ protease inhibitor. Cytoplasmic fractions were prepared by adding TCA to 10% and spinning (21,000 *g* for 5 min at 4°C) after incubation. The pellets were resuspended in RIPA buffer with 1 × HALT protease inhibitor. Biotinylated human-specific anti-FXN antibody was used for immunoprecipitation. The eluate was then separated by SDS-PAGE followed by immunoblotting using an anti-FXN antibody (*see* Supplementary File 4 *for details*).

### PK Calculations and Statistics

PK parameters for nomlabofusp were calculated with the non-compartmental method using Phoenix WinNonlin, version 8.3 or higher (Certara, St. Louis, MO). Allometrically scaled doses were calculated using an allometric exponent approach. Statistical analyses (ANOVA, Student’s t-test, or by Kaplan–Meier Survival curve with log rank analysis) were conducted with GraphPad Prism version 10.0.03 or higher (GraphPad Software, San Diego, CA). Further experiment specific approaches are described in the figure captions. Correlation and regression analyses were conducted using the Statistical Analysis System statistical software package, version 9.4 (SAS Institute Inc., Cary, NC).

## Results

### Nomlabofusp PK is Dose-proportional and Similar Between *Fxn*-KO Mice and WT Animals Following Administration of Allometrically Scaled Doses

**Fig. 1:**
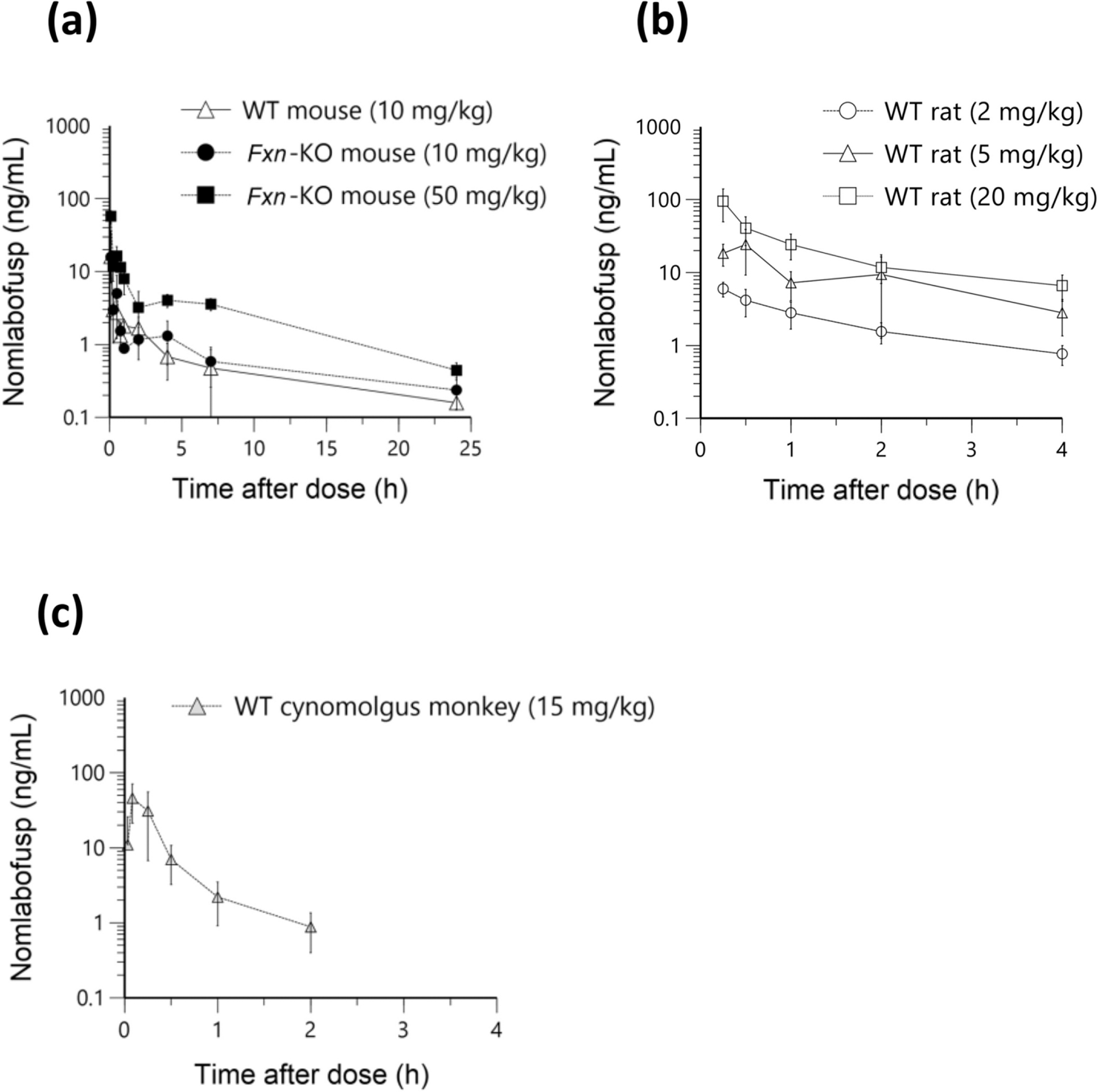
Nomlabofusp Plasma Pharmacokinetics in WT mice and rats, *Fxn*-KO mice and cynomolgus monkeys. Mean (SD) nomlabofusp plasma concentration–time profiles in **a** WT (C57BL6) mice after a single SC 10 mg/kg (*n* = 27 M/0 F) nomlabofusp dose and *Fxn*-KO mice after single SC 10 (*n* = 10 M/5 F) or 50 (*n* = 15 M/12 F) mg/kg nomlabofusp doses (*n* = 3/timepoint/group), samples were quantified using an electrochemiluminescence based immunoassay with an anti-TAT capture antibody; **b** WT Sprague Dawley rats treated daily SC with nomlabofusp 2, 5 or 20 mg/kg. PK samples were collected after 6 days of treatment (*n* = 9 M/timepoint/group), samples were quantified using hybrid LC–MS/MS that is specific to nomlabofusp (anti-TAT antibody capture); **c** Cynomolgus monkeys after a single SC 15 mg/kg nomlabofusp dose (*n* = 3 M/3 F); plasma samples were collected after the first dose and quantified using a qualified ELISA with anti-TAT antibody capture and anti-FXN antibody detection

Following a single 10 mg/kg SC dose of nomlabofusp (equivalent to a 50 mg human dose) administered to C57BL6 WT mice and *Fxn-*KO mice, mean nomlabofusp concentrations were nearly indistinguishable between WT and KO. A higher dose of 50 mg/kg SC in the *Fxn*-KO mice also showed a similar PK profile, with fast SC absorption and elimination, and showed a dose-dependent increase in plasma concentrations (Fig. 1a). As seen in Table I, the 10 mg/kg and 50 mg/kg SC doses in the *Fxn-*KO mice produced approximately dose-proportional C_max_ and AUC_0-last_. Nomlabofusp SC bioavailability was approximately 35%, as determined in WT mice.

Since PK parameters after SC administration of nomlabofusp in WT and KO mice were found to be comparable, we determined that studies in WT animals could be used to further evaluate the PK of nomlabofusp. Thereafter, dosing nomlabofusp daily at 2 mg/kg, 5 mg/kg, or 20 mg/kg in WT sprague dawley rats (equivalent to ~ 25, 50 and 225 mg in human) showed that it was rapidly absorbed after SC administration, with fast elimination and increasing exposure over this dose range (Fig. 1b). PK parameters calculated after the sixth dose are presented in Table II, where Mean C_max_ and AUC_0–last_ appeared dose-proportional.

Likewise, in healthy monkeys dosed with a single 15 mg/kg nomlabofusp SC dose, rapid absorption and elimination phases were observed (Fig. 1c).

### Nomlabofusp Distributes to FRDA-relevant and Peripheral Tissues in Animal Models

**Fig. 2:**
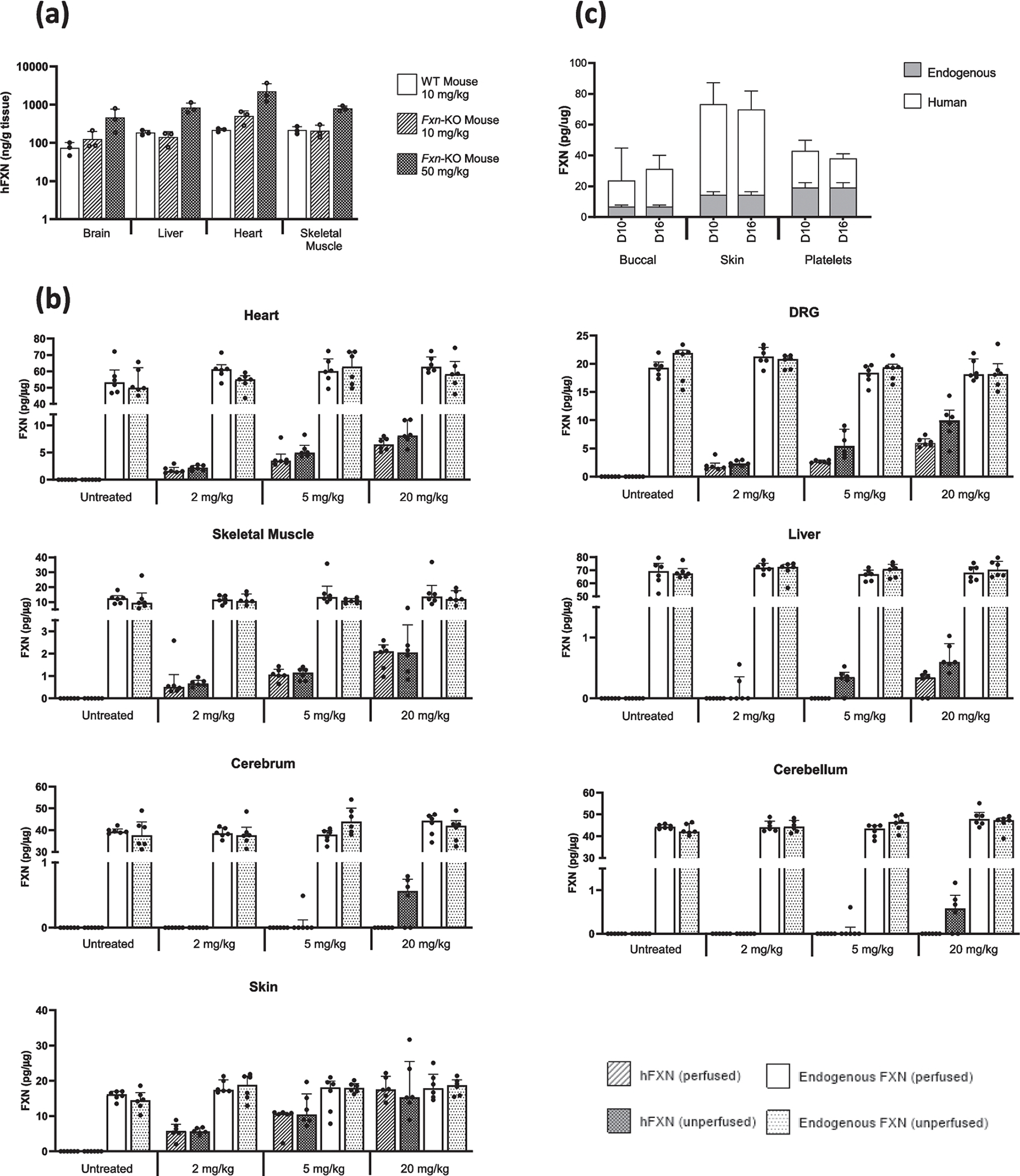
Tissue levels of human FXN after subcutaneous administration of nomlabofusp. **a** Brain, heart, liver and skeletal muscle were harvested 1 h after a single 10 mg/kg SC nomlabofusp dose to wild-type (WT) C57BL6 mice (*n* = 3 M/0 F), a single 10 (*n* = 2 M/1 F) or 50 (*n* = 2 M/1 F) mg/kg dose of nomlabofusp to *Fxn*-KO mice. The tissues were frozen at −80°C and homogenized in RIPA buffer. FXN was quantified using hybrid LC–MS/MS that is specific to nomlabofusp (anti-TAT antibody capture). Peptide SGT was used for detection. Results were normalized to tissue mass. Mean and SD are shown; black circles represent data points from individual animals. **b** Wild-type (WT) Sprague Dawley rats (*n* = 12 M/group) were administered 2, 5 or 20 mg/kg nomlabofusp daily for 7 days via SC injection. The untreated group (*n* = 14 M, dose = 0 mg/kg) remained on study for the same duration as the nomlabofusp treated groups. Approximately 2.5 h after the 7 th dose, half of the animals in each dose group were perfused with PBS and tissues harvested. FXN was quantified using hybrid LC–MS/MS (anti-FXN antibody capture). Peptide SGT was used for detection. Results were normalized to the total tissue protein content of the sample and expressed in pg/μg. Median tissue concentrations and IQR for hFXN or endogenous (rat) FXN in unperfused (*n* = 6 M/dose group) and perfused (*n* = 6 M/dose group) animals in the dorsal root ganglion, skeletal muscle, liver, cerebellum, cerebrum and skin are shown. Black circles represent data points from individual animals. **c** Healthy cynomolgus monkeys were administered 15 mg/kg nomlabofusp twice daily for 14 days starting on Study Day 3. Each animal served as its own control by receiving vehicle for two days before receiving nomlabofusp. On Study Days 10 and 16, tissue collections included whole blood for platelets, skin punch biopsies and buccal swabs. Tissue and cellular homogenates were prepared and hFXN determined using hybrid LC–MS/MS (anti-FXN antibody capture). Peptides SGT and LGG were used for detection. Results were normalized to the total tissue protein content of the sample and expressed in pg/μg. Human FXN values in buccal (*n* = 3 M/3 F), platelets (*n* = 3 M/3 F) and skin (*n* = 3 M/3 F) from the vehicle treated monkeys were below the level of quantification (BLQ). Mean and SD are shown. *Refer to* Supplementary File 2 *for assay schematics (specific peptides)*

WT C57BL6 and *Fxn-*KO mice were administered a single SC dose of nomlabofusp at 10 mg/kg or 50 mg/kg. Brain, heart, liver, and skeletal muscle samples were harvested 1 h after dosing and flash frozen, which were later processed into tissue extracts. In all these samples hFXN concentration was quantified using a hybrid LC–MS/MS assay that excluded the detection of endogenous mouse FXN (*see* Supplementary File 2 *for assay schematics*). As shown in Fig. 2a, hFXN concentrations were comparable between C57BL6 and *Fxn-*KO mice in all 4 tissues, with dose-dependent increase in hFXN after the 50 mg/kg nomlabofusp treatment in the *Fxn-*KO mice, supporting the use of WT animals to evaluate the pharmacodynamics (PD) of nomlabofusp. In the brain, hFXN was quantifiable in concentrations that were higher than the simultaneous plasma concentrations, leading to a brain-to-plasma concentration ratio > 0.04 (25), demonstrating hFXN penetration to brain above any potential contribution from blood contamination.

To understand nomlabofusp exposure and FXN distribution in tissues after multiple doses, WT Sprague Dawley rats were administered 2 mg/kg, 5 mg/kg, or 20 mg/kg nomlabofusp SC daily for 7 days. After the final dose the cerebellum, cerebrum, DRG, liver, heart, skeletal muscle, and skin were collected and tissue homogenates prepared. Endogenous rat FXN and hFXN were quantified by a hybrid LC–MS/MS assay capable of differentiating species-specific FXN. In this experiment, whole-body perfused rats (prior to tissue harvest) were also included to ensure that the observed tissue concentrations would exclude contamination by blood. As seen in Fig. 2b, perfusion had no effect on the endogenous levels of FXN, as would be expected. Across doses in all the rats, the median rat FXN concentrations were 19.3, 60.5, 70.0, 12.2, 17.1, 44.7, and 39.1 pg/μg in perfused DRG, heart, liver, skeletal muscle, skin, cerebellum, and cerebrum, respectively, indicating that endogenous FXN levels are tissue specific. In unperfused rats, nomlabofusp-derived hFXN was measurable in these tissues, showing a dose-dependent increase. Also, in tissues from unperfused rats hFXN concentrations were higher compared with DRG, heart and liver from perfused rats and were not quantifiable in the cerebrum and cerebellum of perfused rats. In skeletal muscle and skin negligible differences were observed in perfused *versus* unperfused samples.

**Fig. 3:**
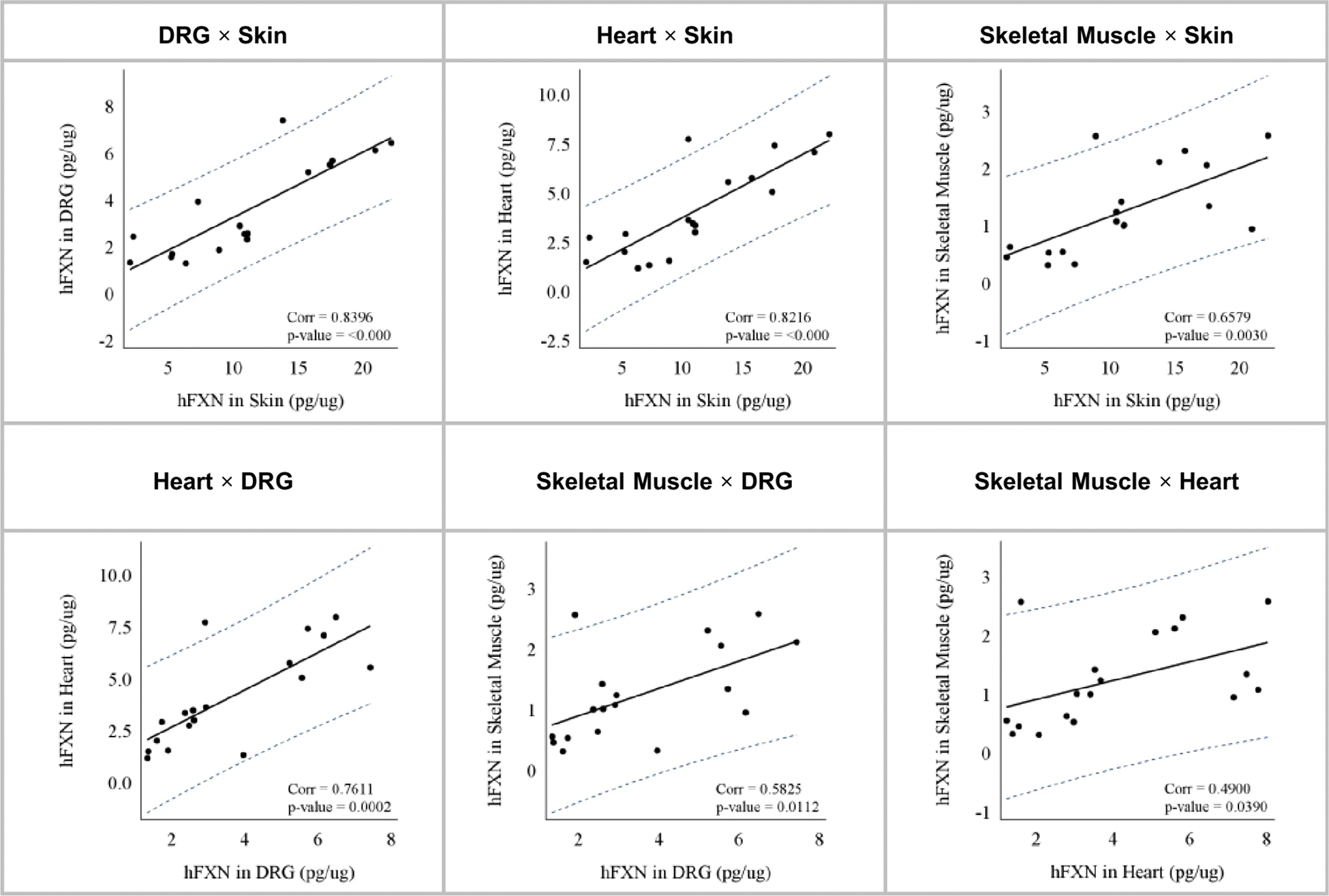
Tissue levels of human FXN amongst various tissues after subcutaneous administration of nomlabofusp in rats. WT rats (*n* = 12 M/group) were dosed SC with 2, 5 or 20 mg/kg nomlabofusp daily for 7 days. Approximately 2.5 h after the 7 th dose, half of each dose group (*n* = 6 M) were perfused and organs harvested. Human FXN in each tissue was quantified using hybrid LC–MS/MS (anti-FXN antibody capture, peptide SGT for detection).and was normalized to the total tissue protein content of the sample and expressed in pg/μg. Comparisons of the concentrations of hFXN in various perfused tissues are shown with Pearson correlation coefficient (Corr) and significance (p). Refer to Supplementary File 2 for assay schematics (specific peptides)

Significant correlations between hFXN in the tissues were also observed, as shown in Fig. 3 (heart/DRG, r= 0.76; skeletal muscle/DRG, r= 0.58; heart/skeletal muscle, r= 0.49). Notably, hFXN increases after nomlabofusp administration in these primary target organs correlated with hFXN increases in the skin, a peripheral tissue (skin/heart, r = 0.82; skin/DRG, r = 0.84; skin/skeletal muscle, r= 0.66). Skin showed the greatest hFXN increase in 7 days (Fig. 2b) and it correlated with the hFXN levels in other organs (Fig. 3), suggesting that skin is a predictor of nomlabofusp penetration into FRDA-relevant tissues. A tissue plasma correlation was also observed (*see* Supplementary File 5), where hFXN increases in tissues correlated with nomlabofusp concentration in plasma at the time of tissue collections.

A longer multiple dose study was conducted in healthy monkeys in which nomlabofusp was administered twice a day SC at 15 mg/kg daily for 14 days (Study Days 3 through 16). Each monkey served as its own control by receiving vehicle alone for 2 days prior to receiving nomlabofusp (Study Days 1 and 2). Tissue samples (buccal swab, skin biopsy, and platelets) were collected on Days 3 (pre-dose), 10, and 16 of nomlabofusp treatment and processed into homogenates in which hFXN, monkey FXN, and nomlabofusp were quantified using hybrid LC–MS/MS. Endogenous monkey FXN was quantifiable in all pre-dose samples. As shown in Fig. 2c, hFXN was present in buccal cells, platelets, and skin as early as Day 10 and had similar levels on Day 16. A small fraction of nomlabofusp was detected in buccal (< 25%) and skin (< 5%) but not in platelets compared to hFXN (*see* Supplementary File 6). Using a nomlabofusp-specific ELISA, nomlabofusp was not detected in any of the CSF samples. However, in the same monkeys, CSF, collected at pre-dose (Study Day 3) and at necropsy (Study Day 16), was tested with another ELISA that detected immunoreactive hFXN (present in both hFXN and nomlabofusp). With this method, signal was quantifiable in all post-treatment samples at concentrations ranging from 1.31 to 5.52 ng/mL, indicating that hFXN was present in the CSF (*see* Supplementary File 6).

### Nomlabofusp Produces Dose-dependent Increases of hFXN in Mitochondria and is Processed to Mature hFXN in Rodents

**Fig. 4:**
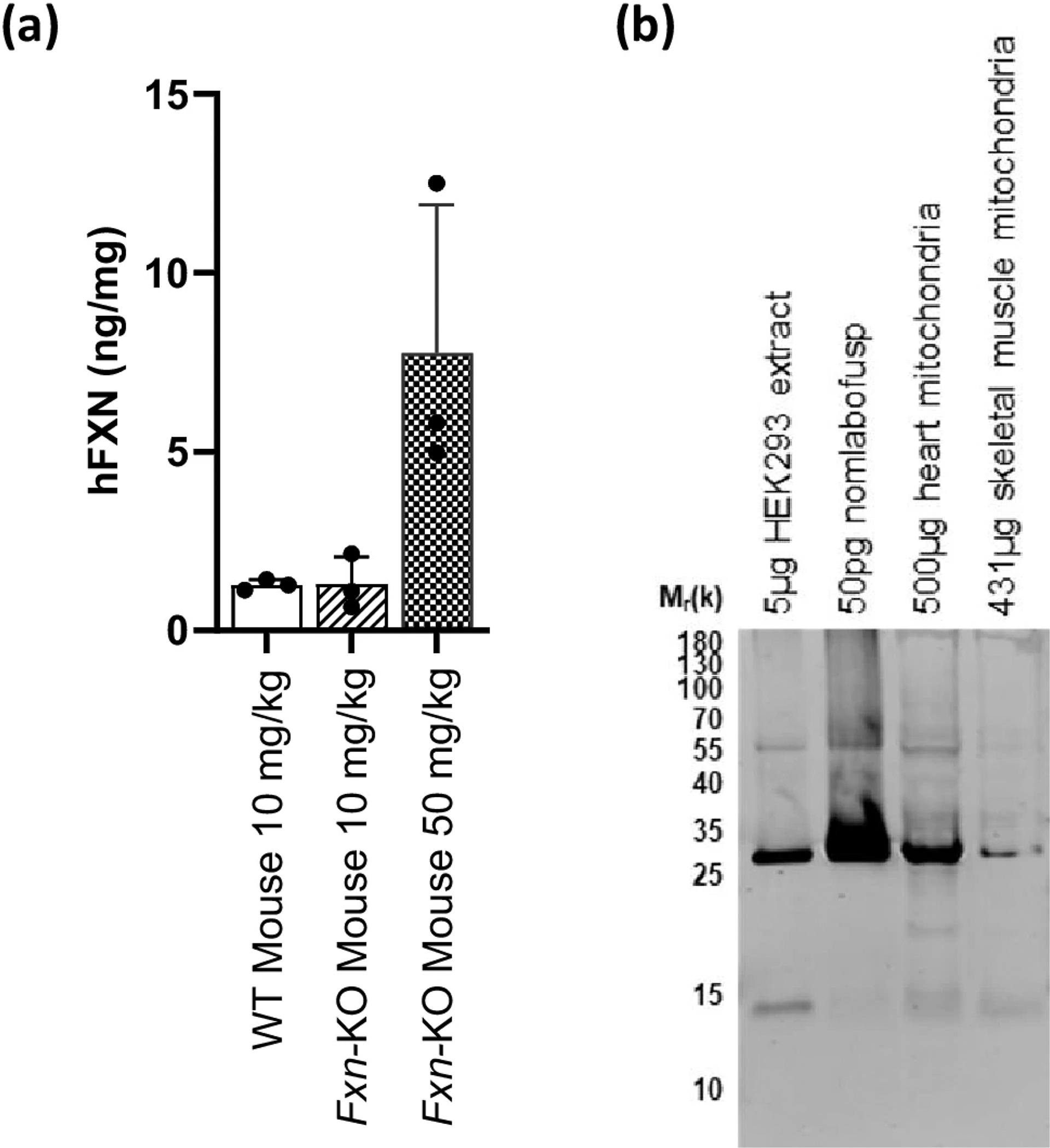
Human FXN in mitochondria after subcutaneous administration of nomlabofusp. **a** Liver was harvested 1 h after a single 10 mg/kg SC nomlabofusp dose to wild-type (WT) C57BL6 mice (*n* = 3 M/0 F), a single 10 (*n* = 2 M/1 F) or 50 (*n* = 2 M/1 F) mg/kg dose of nomlabofusp to *Fxn*-KO mice. Liver mitochondria extracts (“Liver ME”) were prepared on day of harvest using fresh tissue. Human FXN concentrations were quantified using hybrid LC–MS/MS (anti-FXN antibody capture, peptide SGT for detection). Liver ME hFXN concentrations were normalized to the total tissue protein content measured using a BCA assay (ng/mg). Mean and SD are shown; black circles represent data points from individual animals. Refer to Supplementary File 2 for assay schematics (specific peptides). **b** WT rats (*n* = 5 M/group) were administered 20 mg/kg nomlabofusp daily for 7 days via SC injection. Approximately 2.5 h after the 7 th dose animals were perfused with PBS and tissues harvested. Mitochondria were isolated from the heart and skeletal muscle. All samples from each tissue were pooled. FXN was immunoprecipitated from 500 μg total protein from heart mitochondria and 431 μg total protein from skeletal muscle mitochondria using a human-specific FXN antibody and analyzed by western blotting. Nomlabofusp and HEK293 cell extracts were immunoprecipitated as controls to identify the migration of full length nomlabofusp and mature hFXN, respectively

In the WT and *Fxn*-KO mice that were administered a single SC dose of nomlabofusp at 10 mg/kg or 50 mg/kg, liver mitochondria were harvested 1 h after dosing and processed to obtain ME. Human FXN was quantified using a hybrid LC–MS/MS assay. As shown in Fig. 4a, hFXN concentrations in liver ME were comparable between C57BL6 and *Fxn-*KO mice and had a dose-dependent increase.

Having demonstrated that nomlabofusp delivered hFXN into liver mitochondria, it became pertinent to determine whether intramitochondrial hFXN had been processed into mature hFXN within FRDA-relevant tissues. From whole-body perfused rats dosed with 20 mg/kg nomlabofusp, heart and skeletal muscle whole cell homogenates were used to isolate mitochondria, which were then lysed and their extracts collected. For each tissue, ME samples from all rats were pooled and FXN was immunoprecipitated using a hFXN-specific antibody, followed by SDS-PAGE based separation and western blot analysis (Fig. 4b). The band between 25–30 kDa is where nomlabofusp migrates on SDS-PAGE. HEK293 cells demonstrate the presence of mature hFXN (14.3 kDa), as well as endogenous immature/unprocessed hFXN (23.1 kDa), which migrates with nomlabofusp on the blot due to their minor molecular weight difference. The presence of 14.3 kDa bands in heart and skeletal muscle ME were consistent with processed, mature FXN. This result in animals confirms previously published findings, where the presence of mature hFXN was demonstrated in nomlabofusp exposed cell lines and buccal tissue of nomlabofusp dosed patients with FRDA (19). Interestingly, a band consistent with the ~ 17 kDa intermediate form (FXN_56–210_), was also clearly visible in the heart, and less so in skeletal muscle.

### Nomlabofusp Restores SDH Activity in *Fxn*-KO Mice

**Fig. 5:**
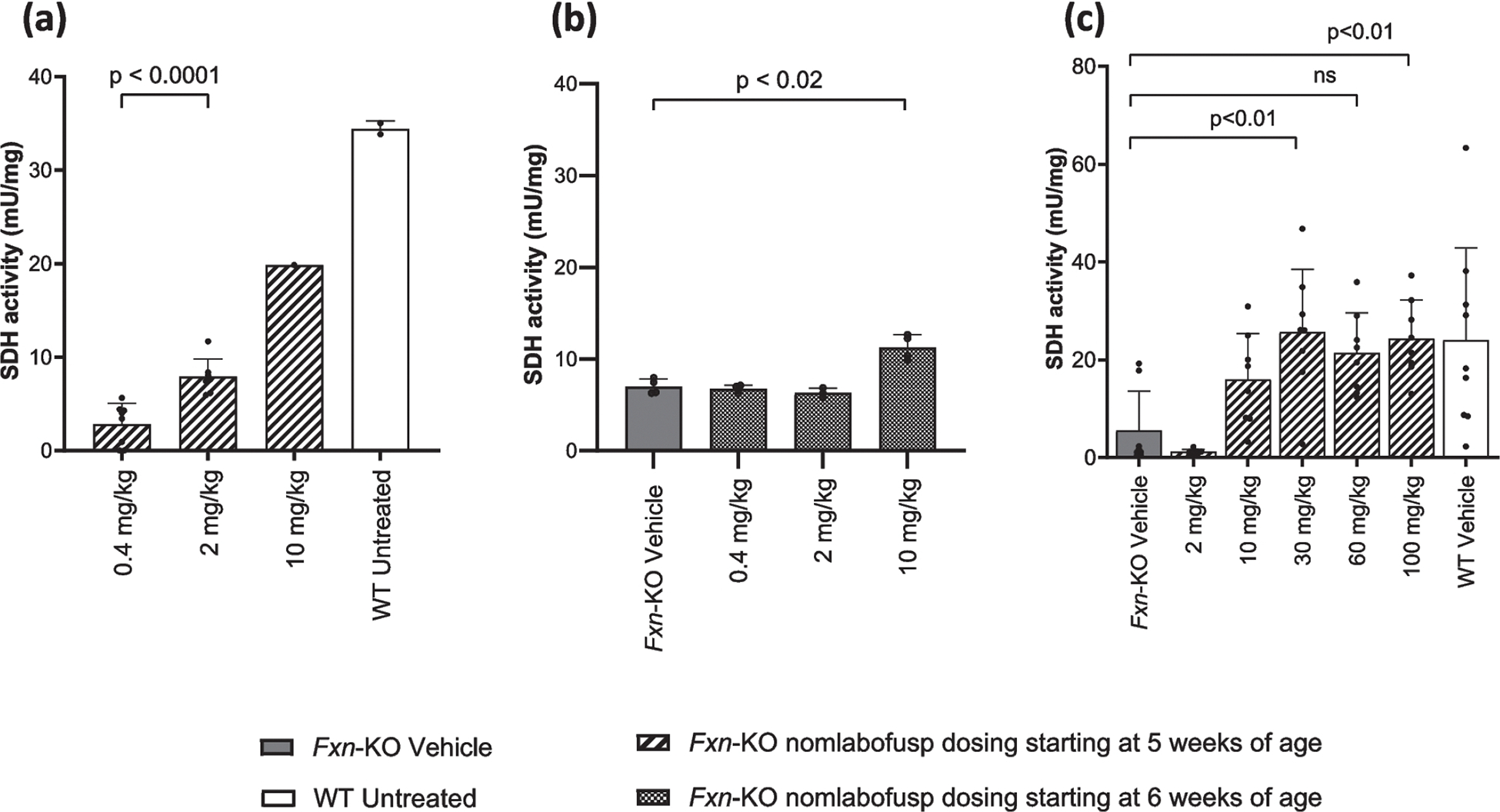
SDH activity after SC administration of nomlabofusp in WT and *Fxn*-KO mice. **a** Heart: Starting at 5 weeks of age *Fxn*-KO mice were administered nomlabofusp at 0.4 (*n* = 4 M/4 F), 2 (*n* = 3 M/4 F) or 10 (*n* = 1 M) mg/kg SC every 48 h for 14 days. After the last dose hearts were harvested at necropsy, and mitochondrial extracts were prepared. SDH activity was measured in heart mitochondrial extracts and mean and SD are shown; black circles represent data points from individual animals. Two-way ANOVA was performed. **b** Heart: Starting at 6 weeks of age *Fxn*-KO mice were administered nomlabofusp at 0.4 (*n* = 2 M/2 F), 2 (*n* = 2 M/1 F), 10 (*n* = 2 M/2 F) mg/kg or vehicle SC daily (*n* = 2 M/2 F) for 20 days. After the last dose hearts were harvested at necropsy, and mitochondrial extracts were prepared. SDH activity was measured in heart mitochondrial extracts and mean and SD are shown; black circles represent data points from individual animals. **c** Skeletal Muscle: *Fxn*-KO mice were treated SC every 48 h for 14 days with 2, 10, 30, 60 or 100 mg/kg (*n* = 4 M/4 F/group) nomlabofusp or vehicle (*n* = 4 M/4 F). WT mice (*n* = 6 M/6 F) were treated SC every 48 h for 14 days with vehicle. After the last dose, skeletal muscle tissue was harvested at necropsy and mitochondrial extracts were prepared. SDH activity was measured in skeletal muscle mitochondrial extracts and mean and SD are shown; black circles represent data points from individual animals. Two-way ANOVA was performed with post-hoc (Tukey) comparison of the parameters

*Fxn-*KO mice were administered nomlabofusp at 5 or 6 weeks of age, when the impact of FXN deficiency had begun to manifest via early signs of progressive cardiomyopathy. As shown in Fig. 5a, nomlabofusp significantly increased SDH activity in heart mitochondria of the *Fxn-*KO mice when dosed every other day for 14 days starting at 5 weeks of age or daily for 20 days starting at 6 weeks of age. Dose-dependent increases were evident between 0.4 mg/kg and 2 mg/kg (*p* < 0.0001) and between the 2 mg/kg and 10 mg/kg dose groups. When mice began receiving nomlabofusp at the age of 6 weeks (Fig. 5b), the 0.4 and 2 mg/kg groups were not statistically different than vehicle treated *Fxn-*KO mice, but the 10 mg/kg/day dose resulted in a significant (*p* < 0.02) increase in SDH activity compared to vehicle treated *Fxn-*KO mice.

Nomlabofusp significantly increased SDH activity in skeletal muscle mitochondria of the *Fxn-*KO treated mice when dosed every other day for 14 days starting at 5 weeks of age (Fig. 5c). The 2 mg/kg dose seemed ineffective, whereas a notable but not statistically significant increase was seen at 10 mg/kg (*p* = 0.49). Nevertheless, significant (*p* < 0.01) increases relative to vehicle treated *Fxn-*KO mice were evident at 30 mg/kg and 100 mg/kg, reaching levels that appeared comparable to WT mice suggesting a plateau effect had been reached.

### Nomlabofusp Treatment Halts Cardiac Dysfunction in *Fxn*-KO Mice

**Fig. 6:**
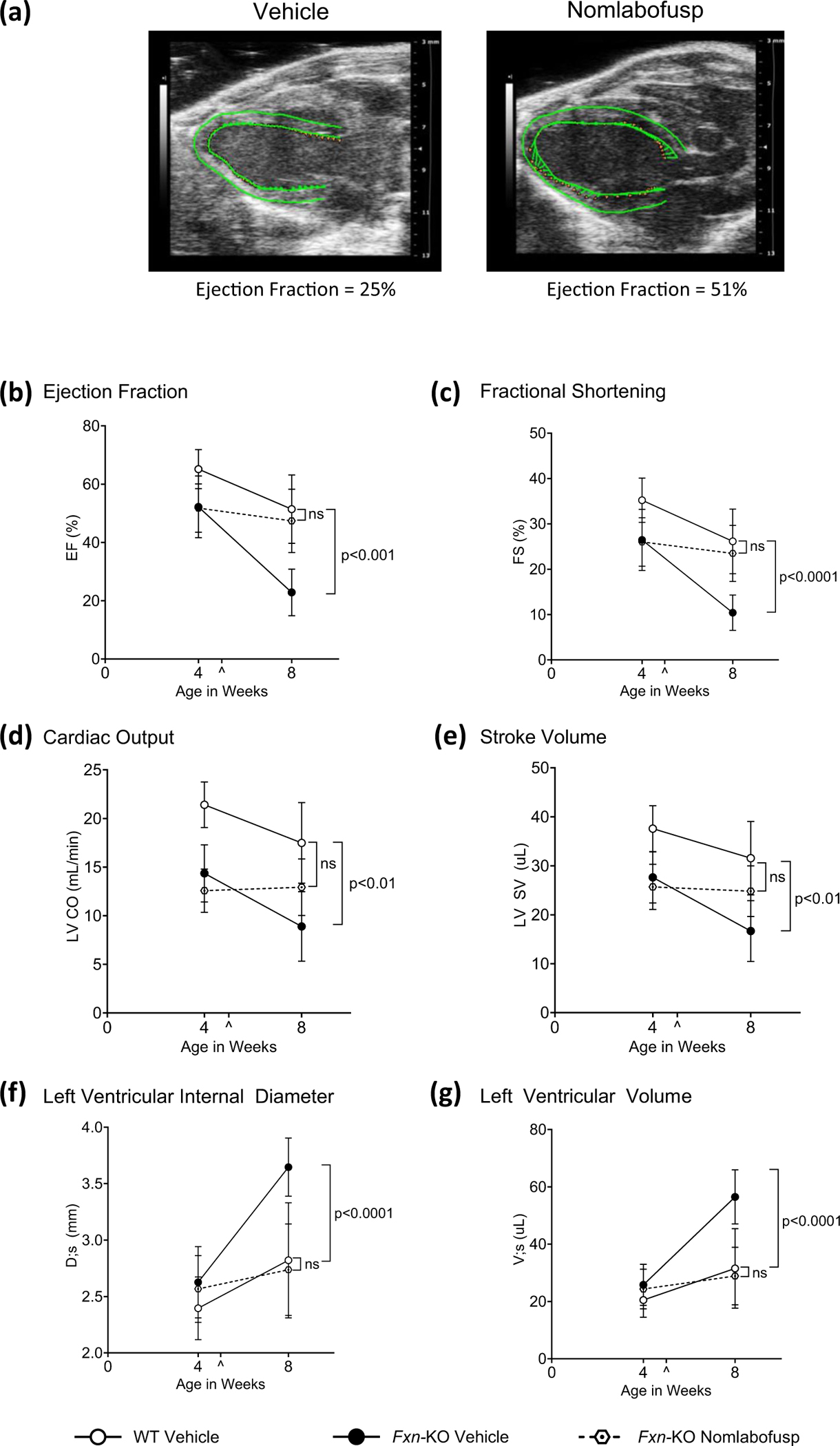
Cardiac function in WT and *Fxn*-KO mice after subcutaneous administration of nomlabofusp. WT or 5-week-old *Fxn*-KO (*n* = 4 M/4 F/group) were treated with either vehicle or nomlabofusp SC at 10 mg/kg every 48 h for 6 weeks. Ultrasound echocardiography assessments were completed at 4 (pre-dose) and 8 weeks of age. **a** Heart echocardiograph images were captured at 8 weeks of age and ejection fraction and global longitudinal strain quantified. Cardiac echocardiography parameters were determined: **b** Left Ventricle Ejection Fraction (EF, %), **c** Fractional Shortening (FS, %), **d** Left Ventricular Cardiac Output (CO, mL/min), **e** Left Ventricle Stroke Volume (SV, μl), **f** Left Ventricle Internal Dimension (D;s, mm), and **g** Left Ventricle Volume, systole (V;s, μl). Mean and SD are shown. Initiation of dosing at 5 weeks of age is denoted by a caret (^). Two-way ANOVA was performed with post-hoc (Tukey) comparison of the parameters. Statistical differences between vehicle treated WT and *Fxn*-KO mice or nomlabofusp treated *Fxn*-KO mice and vehicle treated WT mice are shown

*Fxn-*KO or WT mice were treated SC with 10 mg/kg nomlabofusp or vehicle every other day for 6 weeks, starting at 5 weeks through 11 weeks of age. Baseline cardiac performance was determined by echocardiography at 4 weeks of age prior to the first dose, and post-treatment assessment was done after the mice were 8 weeks of age. Imaging data are shown from representative mice in Fig. 6a. The green demarcated areas represent the left ventricular walls for strain analysis in the vehicle and nomlabofusp treated mice at 8 weeks of age, respectively.

In *Fxn-*KO mice disease onset was demonstrated at 4 weeks of age by the presence of compromised cardiac function measured by 4 parameters that were significantly different compared with WT mice: ejection fraction (*p* < 0.002), fractional shortening (*p* < 0.007), cardiac output (*p* < 0.0001) and stroke volume (*p* < 0.0008), as shown in Fig. 6b–e. In vehicle treated *Fxn*-KO mice cardiac function continued to decline as the mice aged, with further worsening at 8 weeks of age in all 4 parameters observed with statistically significant differences *versus* WT: ejection fraction (*p* < 0.001), fractional shortening (*p* < 0.0001), cardiac output (*p* < 0.01), and stroke volume (*p* < 0.01) (Fig. 6b–e). In contrast, in the *Fxn-*KO mice treated with nomlabofusp these parameters did not decline further by 8 weeks of age and were comparable to WT vehicle treated mice (Fig. 6b–e; statistically insignificant difference with any of the cardiac functional parameters measured). At 4 weeks of age, the decline in cardiac functional output between the study groups was not predicted by alterations in left ventricle size, as neither left ventricle interior dimension (LVID) nor left ventricle volume (LV Vol) were significantly different (Fig. 6f and g). However, by 8 weeks of age, the vehicle treated *Fxn-*KO mice demonstrated significant differences in both left ventricle parameters when compared to vehicle treated WT mice. Left ventricular internal diameter was significantly increased (*p* = 0.0001), as was LV Vol (*p* < 0.001) when compared to WT mice (Fig. 6f and g). Importantly, nomlabofusp treatment prevented the decline in left ventricular parameters, resulting in no significant difference between WT and treated *Fxn-*KO mice by 8 weeks of age (Fig. 6f and g).

Collectively, the data in Fig. 6 demonstrated that delivering exogenous FXN via nomlabofusp administration in these mice prevented further decline in cardiac function and for some outcome measures, such as ejection fraction, the function was comparable to age-matched vehicle treated WT mice.

### Nomlabofusp Treatment Leads to Improved Survival of *Fxn*-KO Mice

**Fig. 7:**
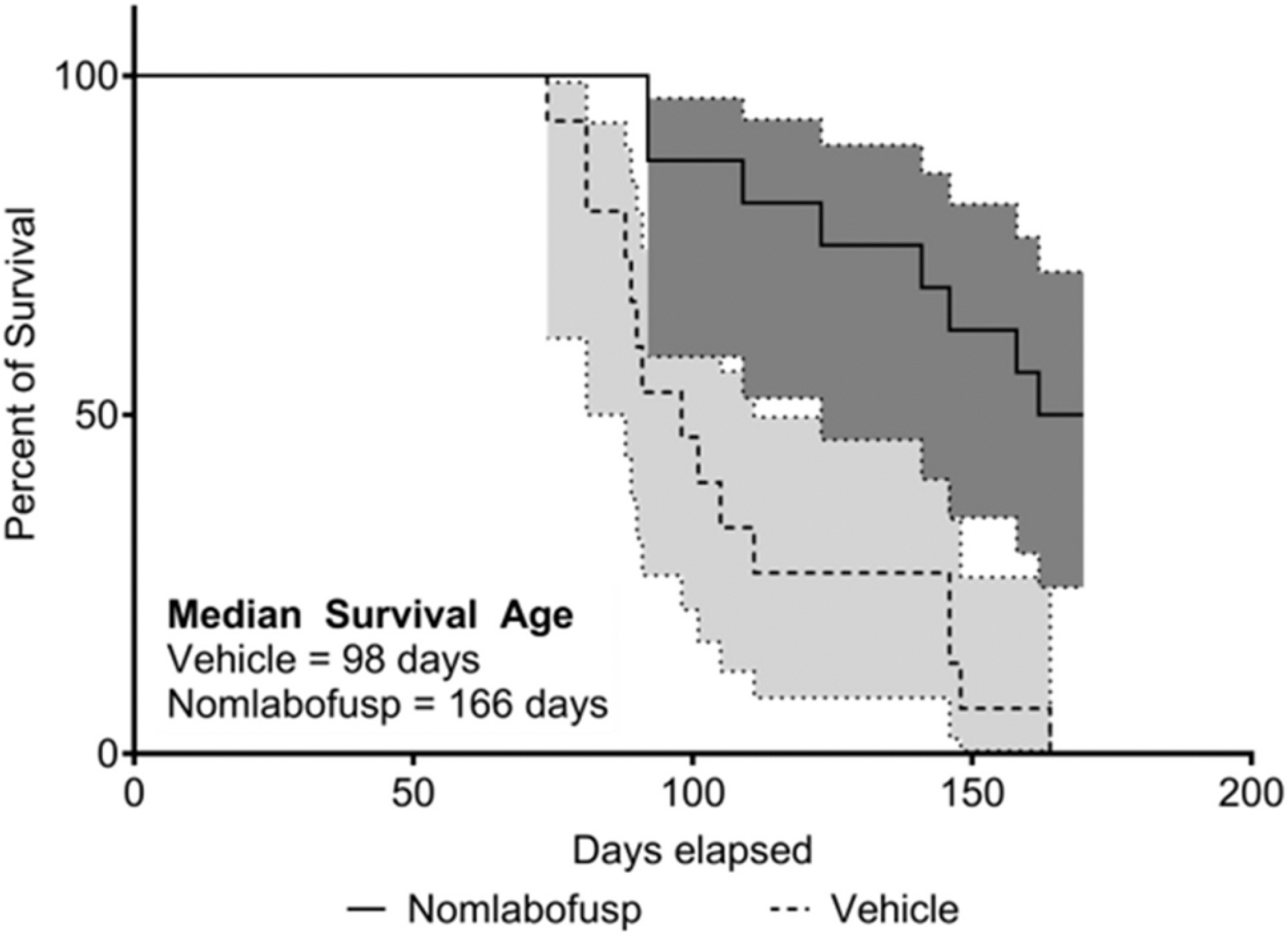
Survival in *Fxn*-KO mice after treatment with nomlabofusp. *Fxn-*KO mice entered the study at 14–16 days of age and were treated with 10 mg/kg nomlabofusp (*n* = 8 M/8 F) or vehicle (*n* = 8 M/8 F) SC 3 times per week up to 170 days of life, until they died, or were removed from the study by 200 days. Data were analyzed by Kaplan–Meier Survival curve with log rank analysis (*p* = 0.0001)

As shown in Fig. 7, *Fxn-*KO mice treated SC with nomlabofusp, 10 mg/kg every other day starting at 2 weeks of age, lived significantly longer than mice treated with vehicle (log rank analysis, *p* < 0.0001). The median survival for mice treated with nomlabofusp was 166 days, compared to 98 days for vehicle-treated mice. At the end of the study period (170 days), none of the vehicle treated mice (*n* = 15) were alive while 8 of the 16 nomlabofusp treated mice were alive. Of the 8 that remained alive, 3 were male and 5 were female, indicating that there was no evidence of sex difference in survival.

## Discussion

In this work we aimed to characterize the PK/PD of nomlabofusp in animals, including the dynamics of endogenous (rodent) FXN and exogenously administered (human) FXN, and evaluate its potential to alter disease progression in humans. We sought to evaluate these aspects in WT rodents and monkeys, and by using the murine striated muscle *Fxn-*KO mouse model, which exhibits a deficiency of FXN protein in the heart and skeletal muscles. The *Fxn*-KO mouse presents a phenotype of progressive weight loss and cardiomyopathy associated with functional cardiac deficits, such as decreased heart rate, decreased ejection fraction, fractional shortening, and increased heart left ventricular mass, ultimately leading to premature death at 65–105 days of age (12). This mouse model has been extensively researched because it represents key aspects of cardiac defects in patients with FRDA (12, 14, 26). The work presented aimed to study potentially therapeutic increases in FXN that are well below the fold-increases in FXN reported by others to be toxic (16, 27).

After SC dosing with nomlabofusp, we observed similar PK properties in WT mice, *Fxn*-KO mice, WT rats, and WT monkeys, which was characterized by rapid absorption, dose-dependent pharmacokinetics, and short half-life. Mice receiving 10 mg/kg and rats receiving 5 mg/kg doses of nomlabofusp had a dose that allometrically scales to ~ 50 mg in humans. These doses in animals led to comparable plasma exposure observed in patients with FRDA who received doses in the intended therapeutic range (25–50 mg) in clinical studies (20). In the same animals, we also observed dose-dependent increases of hFXN in brain, heart, skeletal muscle, liver, and liver mitochondria. The increased FXN levels seen in liver ME of mice treated with 10 mg/kg of nomlabofusp (1–2 ng/mg 1 h post-dose) and restoration of SDH activity were comparable to previously reported findings. Britti *et al.* demonstrated that *Fxn*-KO mice receiving 10 mg/kg of TAT-MTScs-FXN twice weekly achieved FXN concentrations of 1.5 ng/mg of total protein in muscle mitochondrial fractions (15). In their study, when these FXN increases were observed there was also a restoration of SDH activity and increased lifespan, as in our study.

The detection of hFXN in mouse brain at concentrations with a brain to plasma ratio > 0.04 (25), combined with its detection in monkey CSF and perfused rat DRG indicate that nomlabofusp reaches multiple neurological tissues. It is speculated that more sensitive analytical methods, or longer dosing, may lead to a stronger signal in other neurological structures, such as cerebrum and cerebellum, after perfusion.

The FXN concentrations observed in rats showed that changes in tissue hFXN levels after nomlabofusp exposure were consistent across different tissues and highly correlated. The strong correlations between hFXN increases in peripheral tissues, such as skin-DRG, -heart, and -skeletal muscle suggest that peripheral tissues are suitable surrogates for monitoring FXN increases across disease-relevant tissues following nomlabofusp treatment. Importantly, peripheral tissues have been widely used in FRDA natural history studies to measure FXN levels (3, 28, 29).

SDH activity was significantly increased in both heart and skeletal muscle ME from nomlabofusp treated mice compared to untreated or vehicle treated mice. This increase suggests that hFXN delivered by nomlabofusp altered mitochondrial function and demonstrates the functional effect of the delivered hFXN. These changes in tissue mitochondrial activity after nomlabofusp treatment provide evidence that the 69% extension of the lifespan of nomlabofusp treated *Fxn*-KO mice is, at least in part, the result of hFXN-associated improvement in mitochondrial activity. This effect was corroborated by maintenance of cardiac function, including left ventricular dilatation, ejection fraction, stroke volume, and cardiac output observed in nomlabofusp treated *Fxn*-KO mice. Left ventricular dilatation, observed by 8 weeks of age, is considered a compensatory adaptation or consequence of compromised cardiac function (30).

Our findings expand upon previous studies exploring the effect of increasing FXN in similar mouse models. Treatment with a similar TAT-FXN molecule was reported by Vyas *et al.* to increase the mean survival of *Fxn*-KO mice by 50%, accompanied by improved heart rate and cardiac output (31). Using a different molecule, TAT-MTScs-FXN, Britti *et al.* noted a modest increase in lifespan (< 10%) (15). With a gene therapy approach involving an adeno-associated virus (AAV) encoding hFXN, Munoz-Zuluaga *et al*. observed a dose-dependent increase in lifespan of *Fxn*-KO mice (up to 21.5%), and significant improvement in echocardiographic parameters (32). Likewise, Perdomini *et al*. achieved complete correction of left ventricular systolic function and cardiac morphology, along with prolonged survival, using a similar viral vector (17).

Interestingly, we also found that the endogenous FXN level in the heart of WT rats was in the same order of concentration as in humans, indicating consistent FXN levels between species: rat hearts had 54.1 ± 8.35 pg/μg, comparable to the 59 ± 5 pg/μg range reported by Munoz-Zuluaga *et al.* in the hearts of non-FA subjects (32). This cardiac concordance extends to peripheral tissues, where rat skin FXN levels (median 17.1 pg/μg) closely matched human skin measurements (16.4 pg/μg) that we have recently reported (33). In rats we observed median endogenous levels of 44.7, 39.1, and 19.3 pg/μg in cerebellum, cerebrum, and DRG, respectively, whereas FXN levels in WT mice were reported by Gerard et al. to be approximately 40, 60, and 30 pg/μg in the cerebellum, cerebrum, and DRG, respectively (34). In the same order of magnitude were FXN levels reported by Piguet *et al.* in WT mice, approximately 12–25 pg/μg in the DRG and 28 pg/μg in the cerebellum (35). This interspecies consistency in FXN concentrations across cardiac, neural, and dermal tissues reported by multiple laboratories is remarkable. Particularly compelling is the rat-human parity in skin FXN, despite being derived from independent studies using different sampling protocols and detection methods.

The translatability of tissue-specific FXN levels between non-clinical models and humans is critical for evaluating therapeutic interventions. Frataxin concentrations exhibit marked tissue-specific variability, with metabolically active organs like the heart showing substantially higher endogenous levels compared to peripheral tissues (15, 27, 32, 34–37). Our results corroborate that while slight differences were noted in CNS FXN levels between our observations in rats and murine models published elsewhere (34), the overall tissue hierarchy for endogenous FXN concentration (heart > CNS > DRG > skin > muscle) remains consistent. This suggests a fundamental conservation of FXN homeostasis mechanisms across mammalian species. It also validates the utility of rodent models for therapeutic development, particularly when complemented by skin-based monitoring strategies that bridge non-clinical findings with clinical research.

## Conclusion

We demonstrated that the PK of nomlabofusp is comparable between WT rodents and *Fxn*-KO mice. Doses, allometrically scaled to the therapeutic dose range in humans, lead to plasma nomlabofusp concentrations that are similar to nomlabofusp concentrations observed in humans receiving 25 mg and 50 mg nomlabofusp. Nomlabofusp exposure results in a dose-dependent distribution of FXN to clinically accessible peripheral tissues as well as FRDA-associated tissues that is subsequently processed within the mitochondria into mature hFXN. Nomlabofusp treatment reconstituted SDH activity, halted cardiac disease progression, and improved survival in *Fxn*-KO models of FRDA. The consistency between our non-clinical findings and those of other researchers, as well as with our clinical data, underscores the ability of nomlabofusp to increase FXN levels across species, with dose-dependent effects observed in both animal models and patients with FRDA. Collectively, these findings support the potential of nomlabofusp as a novel treatment for adults and children with FRDA that directly addresses FXN deficiency, the root cause of FRDA. Furthermore, our findings validate the translatability of nomlabofusp PK, pharmacology, and FXN tissue distribution data obtained in non-clinical studies to data from clinical studies and provides support for using peripheral tissue FXN levels as a biomarker for potential clinical benefit in adults and children with FRDA.

## Supplementary Material

Suppl 6

Suppl 5

Suppl 4

Suppl 3

Suppl 2

Suppl 1

**Supplementary Information** The online version contains supplementary material available at https://doi.org/10.1208/s12248-025-01093-y.

## Figures and Tables

**Table I:** Summary of nomlabofusp plasma pharmacokinetics in WT and *Fxn*-KO mice. Wild-type (WT) C57BL6 mice were administered a single IV dose of 5 mg/kg (*n* = 24 M/0 F) or single SC dose of 10 mg/kg (*n* = 27 M/0 F) nomlabofusp. *Fxn*-KO mice were administered a single SC dose of either 10 (*n* = 10 M/5 F) or 50 (*n* = 15 M/12 F) mg/kg nomlabofusp (*n* = 3/timepoint/group). Plasma samples were collected at 0.083, 0.25, 0.5, 0.75, 1, 2, 4 and 7 h (IV and SC), and 24 h (SC only) post-dose. Samples were quantified using an electrochemiluminescence based immunoassay. PK parameters were calculated using the mean concentrations at each time point for AUC_last_, area under the plasma concentration *Versus* time curve to the last quantifiable time point; T_max_, time to maximum concentration; C_max_, maximum plasma concentration

	Dose, Route	AUC_last_ (h*ng/mL)	T_max_ (h)	C_max_ (ng/mL)	Bioavailability (%)
WT	5 mg/kg, IV	21.6	-	-	-
	10 mg/kg, SC	15	0.083	16.1	~ 35%
*Fxn*-KO	10 mg/kg, SC	18	0.083	15.9	-
	50 mg/kg, SC	76	0.083	57.7	-

**Table II:** Summary of nomlabofusp plasma pharmacokinetics in WT rats. Wild-type (WT) sprague dawley rats (*n* = 17 M/group) were administered nomlabofusp SC at 2, 5 or 20 mg/kg daily for 7 days. untreated rats (*n* = 14 M) were used as controls. Plasma samples were collected on study day 6 at 0.25, 0.5, 1, 2 and 4 h post-dose and on day 7, 2.5 h post dose (Immediately Prior to Necropsy), and quantified using hybrid LC-MS/MS. Day 6 PK parameters were calculated using the mean profile. AUC_last_, area under the plasma concentration versutime curve to the last quantifiable time point; T_max_, time to maximum concentration; C_max_, maximum plasma concentration

Dose (mg/kg)	Day 6 T_max_ (h)	Day 6 C_max_ (ng/mL)	Day 6 C_max_/Dose (ng/mL/mg/kg)	Day 6 AUC_last_ (h*ng/mL)	Day 6 AUC_last_/Dose (h*ng/mL/mg/kg)	Day 7 2.5 h conc (ng/mL)
2	0.25	5.98	2.99	8.35	4.17	1.63
5	0.50	24.2	4.84	36.4	7.27	4.84
20	0.25	94.9	4.75	82.3	4.11	12.4

## References

[1] Cook A, Giunti P. Friedreich’s ataxia: clinical features, pathogenesis and management. Br Med Bull. 2017;124(1):19–30. 10.1093/bmb/ldx034. 29053830 PMC5862303

[2] Pandolfo M Friedreich Ataxia. Arch Neurol. 2008;75(10):1296–303. 10.1001/archneur.65.10.1296 18852343

[3] Plasterer HL, Deutsch EC, Belmonte M, Egan E, Lynch DR, Rusche JR. Development of frataxin gene expression measures for the evaluation of experimental treatments in friedreich’s ataxia. PLoS ONE. 2013;8(5):e63958. 10.1371/journal.pone.0063958. 23691127 PMC3656936

[4] Campuzano V, Montermini L, Lutz Y, Cova L, Hindelang C, Jiralerspong S, Frataxin is reduced in Friedreich ataxia patients and is associated with mitochondrial membranes. Human Mol Gen. 1997;6(11):1771–80. 10.1093/hmg/6.11.1771 9302253

[5] Gibson TJ, Koonin EV, Musco G, Pastore A, Bork P. Friedreich’s ataxia protein: phylogenetic evidence for mitochondrial dysfunction. Trends Neurosci. 1996;19(11):465–8. 10.1016/S0166-2236(96)20054-2. 8931268

[6] Goetzman E, Gong Z, Zhang B, Muzumdar R. Complex II biology in aging, health, and disease. Antioxidants (Basel). 2023;12(7):1477. 10.3390/antiox12071477. 37508015 PMC10376733

[7] Gonzalez-Cabo P, Vazquez-Manrique RP, Garcia-Gimeno MA, Sanz P, Palau F. Frataxin interacts functionally with mitochondrial electron transport chain proteins. Human Mol Gen. 2005;14(15):2091–8. 10.1093/hmg/ddi214. 15961414

[8] Busi MV, Gomez-Casati DF. Exploring frataxin function. IUBMB Life. 2012;64(1):56–63. 10.1002/iub.577. 22095894

[9] Cossee M, Puccio H, Gansmuller A, Koutnikova H, Dierich A, LeMeur M, Inactivation of the Friedreich ataxia mouse gene leads to early embryonic lethality without iron accumulation. Hum Mol Genet. 2000;9(8):1219–26. 10.1093/hmg/9.8.1219. 10767347

[10] Perdomini M, Hick A, Puccio H, Pook MA. Animal and cellular models of Friedreich ataxia. J Neurochem. 2013;126(Suppl 1):65–79. 10.1111/jnc.12219. 23859342

[11] Mosbach V, Puccio H. A multiple animal and cellular models approach to study frataxin deficiency in Friedreich Ataxia. Biochim Biophys Acta Mol Cell Res. 2024;1871(7):119809. 10.1016/j.bbamcr.2024.119809. 39134123

[12] Puccio H, Simon D, Cossee M, Criqui-Filipe P, Tiziano F, Melki J, Mouse models for Friedreich ataxia exhibit cardiomyopathy, sensory nerve defect and Fe-S enzyme deficiency followed by intramitochondrial iron deposits. Nat Gen. 2001;27:181–6. 10.1038/84818 11175786

[13] Stram AR, Wagner GR, Fogler BD, Pride PM, Hirschey MD, Payne RM. Progressive mitochondrial protein lysine acetylation and heart failure in a model of Friedreich’s ataxia cardiomyopathy. PLoS ONE. 2017;12(5):e0178354. 10.1371/journal.pone.0178354. 28542596 PMC5444842

[14] Rotig A, De Lonlay P, Chretien D, Foury F, Koenig M, Sidi D, Aconitase and mitochondrial iron–sulphur protein deficiency in Friedreich ataxia. Nat Gen. 1997;17:215–7. 10.1038/ng1097-215 9326946

[15] Britti E, Delaspre F, Feldman A, Osborne M, Greif H, Tamarit J, Frataxin-deficient neurons and mice models of Friedreich ataxia are improved by TAT-MTScs-FXN treatment. J Cell Mol Med. 2018;22(2):834–48. 10.1111/jcmm.13365. 28980774 PMC5783845

[16] Huichalaf C, Perfitt TL, Kuperman A, Gooch R, Kovi RC, Brenneman KA, In vivo overexpression of frataxin causes toxicity mediated by iron-sulfur cluster deficiency. Mol Ther Methods Clin Dev. 2022;24:367–78. 10.1016/j.omtm.2022.02.002. 35252470 PMC8866050

[17] Perdomini M, Belbellaa B, Monassier L, Reutenauer L, Messaddeq N, Cartier N, Prevention and reversal of severe mitochondrial cardiomyopathy by gene therapy in a mouse model of Friedreich’s ataxia. Nat Med. 2014;20(5):542–7. 10.1038/nm.3510. 24705334

[18] Vives E, Brodin P, Lebleu B. A truncated HIV-1 Tat protein basic domain rapidly translocates through the plasma membrane and accumulates in the cell nucleus. J Biol Chem. 1997;272(25):16010–7. 10.1074/jbc.272.25.16010. 9188504

[19] Baile MG, Jones J, Sahr N, Shankar G. Nomlabofusp, a fusion protein of human frataxin and a cell penetrant peptide, delivers mature and functional frataxin into mitochondria. AAPS J. 2025;27(3):68. 10.1208/s12248-025-01054-5. 40140196

[20] Clayton R, Galas T, Scherer N, Farmer J, Ruiz N, Hamdani M, Safety, pharmacokinetics, and pharmacodynamics of nomlabofusp (CTI-1601) in Friedreich’s ataxia. Ann Clin Transl Neurol. 2024;11(3):540–53. 10.1002/acn3.51971. 38311797 PMC10963286

[21] Scherer N, Clements-Egan A, Hamdani M, Shenouda M, Forest A, Des Rosiers C, Effect of nomlabofusp administration on tissue frataxin levels, plasma lipid profiles, and gene expression in patients with Friedreich’s ataxia. London: International Congress for Ataxia Research; 2024.

[22] Frezza C, Cipolat S, Scorrano L. Organelle isolation: functional mitochondria from mouse liver, muscle and cultured fibroblasts. Nat Protoc. 2007;2(2):287–95. 10.1038/nprot.2006.478. 17406588

[23] Del Gaizo V, Payne RM. A novel TAT–Mitochondrial signal sequence fusion protein is processed, stays in mitochondria, and crosses the placenta. Mol Ther. 2003;7(6):720–30. 10.1016/s1525-0016(03)00130-8. 12788645

[24] MacKenzie JA, Payne RM. Ribosomes specifically bind to mammalian mitochondria via protease-sensitive proteins on the outer membrane. J Biol Chem. 2004;279(11):9803–10. 10.1074/jbc.M307167200. 14668341

[25] Shaffer CL. Chapter 4 - Defining neuropharmacokinetic parameters in CNS drug discovery to determine cross-species pharmacologic exposure-response relationships. In: Macor JE, editor. Annual Reports in Medicinal Chemistry. Academic Press; 2010. p. 55–70.

[26] Perfitt TL, Huichalaf C, Gooch R, Kuperman A, Ahn Y, Chen X, A modified mouse model of Friedreich’s ataxia with conditional Fxn allele homozygosity delays onset of cardiomyopathy. Am J Physiol Heart Circ Physiol. 2024;326(2):H357–69. 10.1152/ajpheart.00496.2023. 38038720

[27] Belbellaa B, Reutenauer L, Messaddeq N, Monassier L, Puccio H. High levels of frataxin overexpression leads to mitochondrial and cardiac toxicity in mouse models. BioRxiv. 2020. 10.1101/2020.03.31.015255. PMC7648087 33209958

[28] Sacca F, Puorro G, Antenora A, Marsili A, Denaro A, Piro R, A combined nucleic acid and protein analysis in Friedreich ataxia: implications for diagnosis, pathogenesis and clinical trial design. PLoS ONE. 2011;6(3):e17627. 10.1371/journal.pone.0017627. 21412413 PMC3055871

[29] Willis JH, Isaya G, Gakh O, Capaldi RA, Marusich MF. Lateral-flow immunoassay for the frataxin protein in Friedreich’s ataxia patients and carriers. Mol Genet Metab. 2008;94(4):491–7. 10.1016/j.ymgme.2008.03.019. 18485778 PMC2692602

[30] Huang ML, Sivagurunathan S, Ting S, Jansson PJ, Austin CJ, Kelly M, Molecular and functional alterations in a mouse cardiac model of Friedreich ataxia: activation of the integrated stress response, eIF2alpha phosphorylation, and the induction of downstream targets. Am J Pathol. 2013;183(3):745–57. 10.1016/j.ajpath.2013.05.032. 23886890

[31] Vyas PM, Tomamichel WJ, Pride PM, Babbey CM, Wang Q, Mercier J, A TAT-frataxin fusion protein increases lifespan and cardiac function in a conditional Friedreich’s ataxia mouse model. Hum Mol Genet. 2012;21(6):1230–47. 10.1093/hmg/ddr554. 22113996 PMC3284115

[32] Munoz-Zuluaga C, Gertz M, Yost-Bido M, Greco A, Gorman N, Chen A, Identification of safe and effective intravenous dose of AAVrh10hFXN to treat the cardiac manifestations of friedreich’s ataxia. Hum Gene Ther. 2023;34(13–14):605–15. 10.1089/hum.2023.020. 37166361 PMC10354731

[33] De Toni F, Scherer N, Schapiro E, Shenouda M, Hamdani M, Shankar G, Prediction of tissue frataxin levels with long term administration of nomlabofusp in adults with Friedreich’s ataxia using modeling and simulations. London: International Congress for Ataxia Research; 2024.

[34] Gerard C, Archambault AF, Bouchard C, Tremblay JP. A promising mouse model for Friedreich Ataxia progressing like human patients. Behav Brain Res. 2023;436:114107. 10.1016/j.bbr.2022.114107. 36089099

[35] Piguet F, de Montigny C, Vaucamps N, Reutenauer L, Eisenmann A, Puccio H. Rapid and complete reversal of sensory ataxia by gene therapy in a novel model of friedreich ataxia. Mol Ther. 2018. 10.1016/j.ymthe.2018.05.006. PMC6094869 29853274

[36] Bouchard C, Gerard C, Yanyabe SG, Majeau N, Aloui M, Buisson G, Finding an appropriate mouse model to study the impact of a treatment for friedreich ataxia on the behavioral phenotype. Genes (Basel). 2023;14(8):1654. 10.3390/genes14081654. 37628705 PMC10454134

[37] Nachbauer W, Wanschitz J, Steinkellner H, Eigentler A, Sturm B, Hufler K, Correlation of frataxin content in blood and skeletal muscle endorses frataxin as a biomarker in Friedreich ataxia. Mov Disord. 2011;26(10):1935–8. 10.1002/mds.23789. 21692115

