# Supplementary material for: Pharmacokinetics and Pharmacodynamics of Nomlabofusp in Non-clinical Studies of Friedreich’s Ataxia
Source: AAPS J. Author manuscript; Available in PMC 2026 May 5. (PMC13143400; doi:10.1208/s12248-025-01093-y)
Supplement: Suppl 6 [file NIHMS2151153-supplement-Suppl_6.pdf]

SUPPLEMENTARY FILE 6

Additional tissue penetration data of non-human primates.

Endogenous FXN (normalized LGG) was quantifiable in all buccal, skin and platelets pre-dose samples, as seen in the table below:

Tissue	Animal ID	Day	Normalized LGG (pg/μg)
Buccal	EAA1M001	3	6.65
Buccal	EAA1M002	3	8.07
Buccal	EAA1M003	3	6.35
Buccal	EAA1F004	3	5.44
Buccal	EAA1F005	3	7.90
Buccal	EAA1F006	3	6.36
Skin	EAA1M1	3	16.92
Skin	EAA1M2	3	16.12
Skin	EAA1M3	3	15.91
Skin	EAA1F4	3	12.55
Skin	EAA1F5	3	14.24
Skin	EAA1F6	3	11.76
Platelets	EAA1M001	3	20.35
Platelets	EAA1M002	3	20.62
Platelets	EAA1M003	3	19.06
Platelets	EAA1F004	3	12.58
Platelets	EAA1F005	3	20.97
Platelets	EAA1F006	3	21.06

Nomlabofusp (normalized GGM) was detected in buccal (<25%) and skin (<5%) but not in platelets compared to hFXN (normalized SGT), as seen in the table below:

Tissue	Animal ID	Day	Normalized GGM (pg/μg)	Normalized SGT (pg/μg)	% of GGM to SGT	Average % of GGM to SGT
Buccal	EAA1M001	10	0.809	6.861	11.8	24.2
Buccal	EAA1M002	10	28.606	88.100	32.5	
Buccal	EAA1M003	10	0.645	5.454	11.8	
Buccal	EAA1F004	10	15.063	26.011	57.9	
Buccal	EAA1F005	10	4.305	17.680	24.3	
Buccal	EAA1F006	10	2.216	9.372	23.6	
Buccal	EAA1M001	16	6.101	32.196	18.9	
Buccal	EAA1M002	16	4.363	29.334	14.9	
Buccal	EAA1M003	16	6.484	42.738	15.2	
Buccal	EAA1F004	16	8.721	36.099	24.2	
Buccal	EAA1F005	16	8.159	31.028	26.3	
Buccal	EAA1F006	16	3.504	12.223	28.7	
Platelets	EAA1M001	10	BLQ	36.698		0.0
Platelets	EAA1M002	10	BLQ	24.154		
Platelets	EAA1M003	10	BLQ	24.210		
Platelets	EAA1F004	10	BLQ	20.880		
Platelets	EAA1F005	10	BLQ	20.634		
Platelets	EAA1F006	10	BLQ	16.464		
Platelets	EAA1M001	16	BLQ	22.628		
Platelets	EAA1M002	16	BLQ	16.784		
Platelets	EAA1M003	16	BLQ	13.905		
Platelets	EAA1F004	16	BLQ	20.693		
Platelets	EAA1F005	16	BLQ	18.329		
Platelets	EAA1F006	16	BLQ	20.824		
Skin	EAA1M1	10	1.468	68.701	2.1	4.16
Skin	EAA1M2	10	1.153	56.368	2.0	
Skin	EAA1M3	10	2.272	59.972	3.8	
Skin	EAA1F4	10	2.044	80.631	2.5	
Skin	EAA1F5	10	1.144	37.885	3.0	
Skin	EAA1F6	10	0.987	58.344	1.7	
Skin	EAA1M1	16	3.208	73.019	4.4	
Skin	EAA1M2	16	5.420	50.325	10.8	
Skin	EAA1M3	16	1.896	58.918	3.2	
Skin	EAA1F4	16	3.684	68.048	5.4	
Skin	EAA1F5	16	2.969	40.941	7.3	
Skin	EAA1F6	16	2.206	60.594	3.6	

BLQ: Below limit of quantitation

Nomlabofusp was not found in the CSF samples collected either at Study Day 3 (pre-treatment) or Study Day 16 (post-treatment), as seen in the table below:

Animal ID (Day)	Nomlabofusp Concentration (ng/mL)
EAA1M001 Day 3	BLQ
EAA1F004 Day 3	BLQ
EAA1M001 Day 16	BLQ
EAA1M002 Day 16	BLQ
EAA1M003 Day 16	BLQ
EAA1F004 Day 16	BLQ
EAA1F005 Day 16	BLQ
EAA1F006 Day 16	BLQ

BLQ: Below Limit of Quantitation of 0.800 ng/mL

Immunoreactive hFXN (present in both hFXN and nomlabofusp) was not found in any of the pre-treatment samples (Study Day 3) and in all the post-treatment samples of the CSF samples (Study Day 16), as seen in the table below:

Animal ID (Day)	Immunoreactive hFXN (pg/mL)
EAA1M001 Day 3	BLQ
EAA1F004 Day 3	BLQ
EAA1M001 Day 16	2182
EAA1M002 Day 16	1314
EAA1M003 Day 16	1482
EAA1F004 Day 16	1969
EAA1F005 Day 16	2220
EAA1F006 Day 16	5516*

BLQ (Below Limit of Quantitation) of 9.88 pg/mL

*Sample was contaminated with blood
