# Supplementary material for: Pharmacokinetics and Pharmacodynamics of Nomlabofusp in Non-clinical Studies of Friedreich’s Ataxia
Source: AAPS J. Author manuscript; Available in PMC 2026 May 5. (PMC13143400; doi:10.1208/s12248-025-01093-y)
Supplement: Suppl 5 [file NIHMS2151153-supplement-Suppl_5.pdf]

SUPPLEMENTARY FILE 4

Analysis of mitochondrial fraction using immunoprecipitation and Western Blots was conducted. See the schematic below showing the molecular weights of the various forms of nomlabofusp and FXN that were monitored.

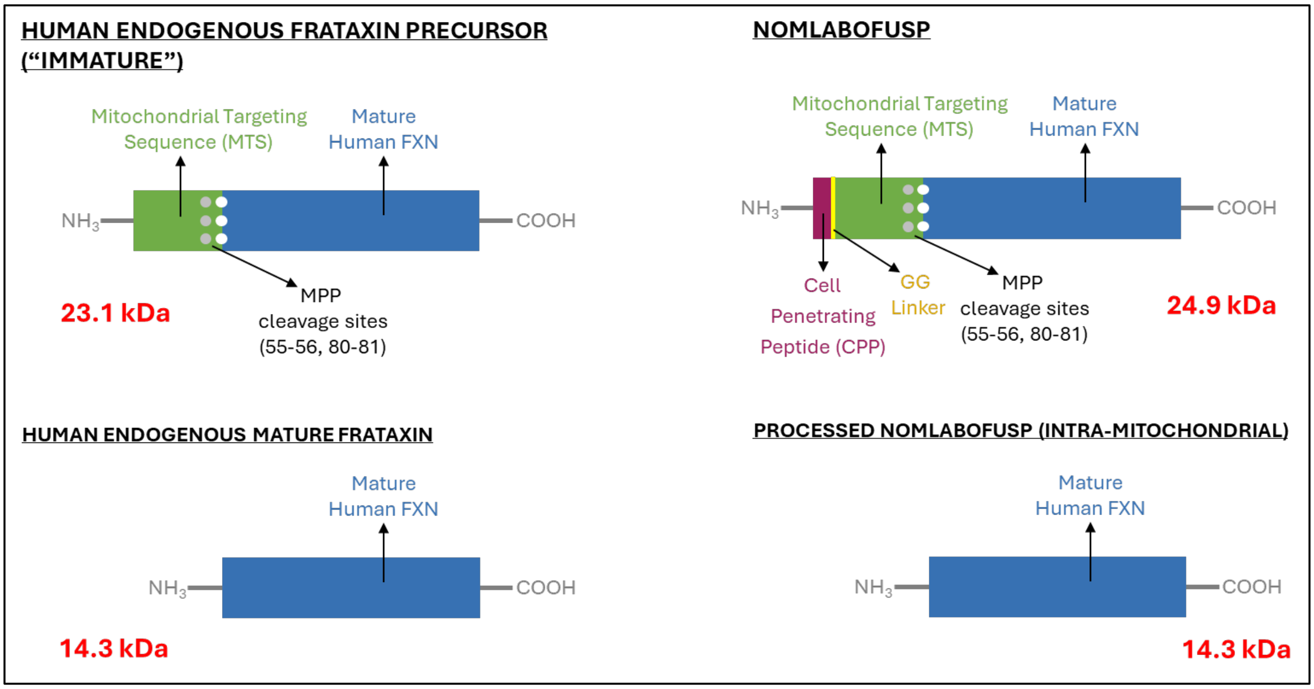

Mitochondrial fractions were thawed on ice, then samples centrifuged at 21,000g for 5 min at 4°C. Supernatant was aspirated, and pelleted mitochondria resuspended in RIPA buffer (Millipore Sigma, R0278) with 1x HALT protease inhibitor (Thermo Fisher Scientific, 78438). Protein concentration was determined using the BCA protein assay kit (Thermo Fisher Scientific, 23225). Cytoplasmic fractions were prepared by adding tricarboxylic acid (TCA) to 10% and incubating them on ice for 30 minutes. Samples were centrifuged at 21,000g for 5 minutes at 4°C, the supernatants removed, and pellets washed with acetone. Samples were centrifuged again at 21,000g for 5 min at 4°C. The acetone supernatant was removed and pellets allowed to partially air dry. 2.5 µL 1M NaOH added to the samples, then pellets resuspended in an equal volume of RIPA buffer with 1x HALT protease inhibitor as the corresponding mitochondrial fraction.

Human-specific anti-FXN antibody (Abcam, ab110328) was biotinylated using the EZ-link Sulfo-NHS-LC-Biotin kit (Thermo Fisher Scientific, A39257). The Sulfo-Biotin solution was diluted to 5.5 mg/mL in PBS and added to 100µg antibody per tube and rotated for 60 minutes at room temperature. After incubation, the solution was further diluted with 100 µL PBS and added to a Zeba column (ThermoFisher Scientific, 89890), which was prepared by washing 3x with PBS. An additional 50 µL of PBS were added to the Zeba column, then centrifuged at 1070g for 2 minutes at room temperature. The flow through (biotinylated antibody) was collected and stored at 4°C until use. 50 µL of streptavidin magnetic beads (Pierce, 88817) per reaction were washed with PBS using magnetic separation, then blocked in Intercept Blocking Buffer for 60 minutes at room temperature while rotating. Beads were washed with PBS, then resuspended in 500 µL PBS. 5 µg biotinylated anti-FXN antibody was added, then incubated for 60 minutes at room temperature while rotating. The conjugated beads were then washed 3x with 1 mL PBS. For immunoprecipitation, conjugated

beads were resuspended in 500 µL PBS, and lysates added. Samples were incubated for 3 hours at room temperature while rotating, then washed 3x with PBS. Bound material was eluted by the addition of 40 µL 1x loading buffer with β-mercaptoethanol and incubating at 80°C for 10 minutes before magnetic separation. The eluate was then analyzed by immunoblotting using an anti-FXN antibody (Abcam, ab219414).

Lysates were diluted to equal concentrations using water, and protein loading buffer with β-mercaptoethanol added according to the manufacturer's instructions. Equal volumes of protein extracts were then loaded onto BOLT 12% Bis-Tris gels (Thermo Fisher Scientific, IB23002) and run in 1x MES SDS running buffer (Invitrogen, B0002). Proteins were transferred onto a nitrocellulose membrane using the iBlot2 at 20 V for 60 seconds, 23 V for 90 seconds, then 25 V for 35 seconds. Membranes were blocked in Intercept blocking buffer for 60 minutes at room temperature. Primary antibodies were diluted in Intercept T20 antibody diluent and incubated on the membranes overnight at 4 °C. The membranes were then washed 3x with TBST (Avantor, Radnor, PA, USA, J640), then incubated with goat anti-mouse 800CW (Li-Cor Biosciences, 925-32210) and goat anti-rabbit 680LT secondary antibodies (Li-Cor Biosciences, 925-68071) diluted in Intercept T20 antibody diluent for 60 minutes at room temperature. Membranes were washed 3x with TBST, then imaged using an Odyssey CLx (Li-Cor Biosciences).
