# Supplementary material for: Pharmacokinetics and Pharmacodynamics of Nomlabofusp in Non-clinical Studies of Friedreich’s Ataxia
Source: AAPS J. Author manuscript; Available in PMC 2026 May 5. (PMC13143400; doi:10.1208/s12248-025-01093-y)

SUPPLEMENTARY FILE 5

Human FXN concentrations (pg/µg) in various organs collected after whole body perfusion as related to simultaneous plasma nomlabofusp concentrations.

Panels show correlations between hFXN levels from Sprague Dawley rats after 7 days of SC treatment with nomlabofusp 2, 5 or 20 mg/kg. (A) Heart (B) DRG (C) Skeletal muscle (D) Skin. Plasma samples were collected 2.5h after the 7th dose and immediately prior to perfusion. Tissues were harvested after perfusion.

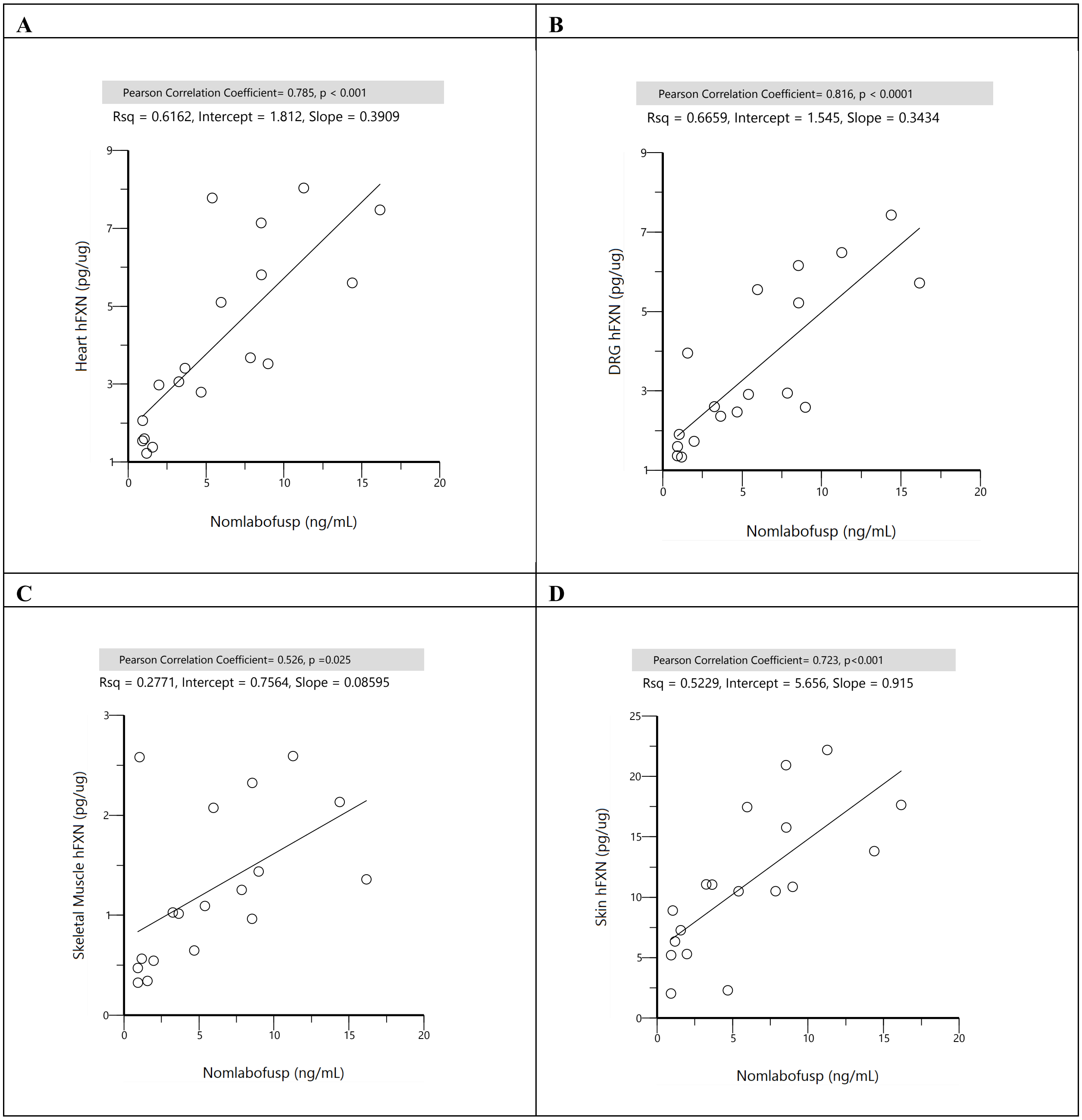

Supplement: Suppl 4 [file NIHMS2151153-supplement-Suppl_4.pdf]
