# Supplementary material for: Pharmacokinetics and Pharmacodynamics of Nomlabofusp in Non-clinical Studies of Friedreich’s Ataxia
Source: AAPS J. Author manuscript; Available in PMC 2026 May 5. (PMC13143400; doi:10.1208/s12248-025-01093-y)
Supplement: Suppl 3 [file NIHMS2151153-supplement-Suppl_3.pdf]

SUPPLEMENTARY FILE 3

Succinate dehydrogenase (SDH) activity was measured in mitochondrial extracts as follows.

Mitochondrial extracts were prepared from flash frozen tissue stored at -80°C using differential centrifugation with commercially available buffers (Abcam, ab110168) or prepared according to previously published methods by the Payne lab [1, 2]. Isolated mitochondria were quantitated using DC Protein Assay Kit (BioRad, 50000112). Skeletal and heart mitochondrial extracts SDH activity were measured using a colorimetric assay kit according to manufacturer's protocol (BioVision, K660-100). Activity was determined by measuring absorbance at 600nm every minute for 30 minutes. Activity was expressed in mU/mg of mitochondrial extract and calculated from a pre-determined time interval. Protein input per well was 5.6 +/- 2.0 µg.

REFERENCES:

1. Del Gaizo V, Payne RM. A novel TAT–Mitochondrial signal sequence fusion protein is processed, stays in mitochondria, and crosses the placenta. Molecular Therapy. 2003;7(6):720-30. doi: 10.1016/s1525-0016(03)00130-8.
2. MacKenzie JA, Payne RM. Ribosomes specifically bind to mammalian mitochondria via protease-sensitive proteins on the outer membrane. J Biol Chem. 2004;279(11):9803-10. doi: 10.1074/jbc.M307167200.
