# Supplementary material for: Pharmacokinetics and Pharmacodynamics of Nomlabofusp in Non-clinical Studies of Friedreich’s Ataxia
Source: AAPS J. Author manuscript; Available in PMC 2026 May 5. (PMC13143400; doi:10.1208/s12248-025-01093-y)
Supplement: Suppl 2 [file NIHMS2151153-supplement-Suppl_2.pdf]

## SUPPLEMENTARY FILE 2

**Bioanalytical procedures for all biological matrices are presented separated by sample preparation and assay quantification methods.**

### Sample preparation

#### *Mouse Plasma*

Blood samples were collected into anti-coagulant $K_2$EDTA tubes pre-loaded with EDTA free Halt™ protease inhibitor cocktail (ThermoFisher) on ice, and centrifuged at 1000*g* for 10 minutes at 4°C. Plasma aliquots were frozen and stored at -80°C.

#### *Mouse Tissue Homogenates*

Whole brain, whole heart, whole liver, and ~ 1 g of skeletal muscle tissues were collected from each animal. After collection, each tissue sample was rinsed with ice-cold saline, and dried on filter paper, then divided into two parts: one was snap frozen for tissue quantification and the second was kept on wet ice for the mitochondrial fraction preparation. All samples (plasma, tissues and mitochondrial fraction samples) were stored at -80°C until processed for analysis. For whole tissue analysis, 200-300 mg of tissue was thawed, suspended in ice-cold PBS pH 7.4 supplemented with EDTA free Halt™ protease inhibitor cocktail and 10mM EDTA and rinsed thoroughly. Rinse was removed, and 2 more washes were performed to remove blood thoroughly. 1mL of radioimmunoassay precipitation buffer (RIPA) buffer (25mM Tris HCl, pH 7.6, 150 mM NaCl, 1% NP-40, 1% sodium deoxycholate, 0.1% SDS) was added per mg tissue and the sample was homogenized using a bead mill. The lysate was adjusted to 1mg/mL with RIPA buffer containing Halt™ protease inhibitor cocktail and 10 mM EDTA, and frozen until analysis.

#### *Mouse Mitochondrial Extracts for the Quantification of FXN*

Fresh liver tissue was rinsed, finely minced and transferred to a dounce tube containing Isolation Buffer for Cells (IBc; 10 mM TrisMOPS, 1mM EGTA/Tris, 200 mM Sucrose, pH 7.4). Each sample was homogenized, transferred to a 1.5 mL Eppendorf tube and centrifuged (10 min at 600 g, 4°C) to pellet cell debris. The supernatant was then transferred to another tube and centrifuged again for 10 min at 7000 g, 4°C to pellet mitochondria, resuspended in 1mL IBc and centrifuged again to re-pellet mitochondria. Supernatant was removed and discarded, and the pellet was homogenized in 200 µL RIPA buffer (150 mM NaCl, 1% NP-40, 1% sodium deoxycholate, 0.1% SDS) containing EDTA free Halt™ protease inhibitor cocktail and 10 mM EDTA, to produce a mitochondrial extract that was frozen at -80°C until analysis.

#### *Rat Plasma*

Plasma samples were collected into $K_2$EDTA tubes and centrifuged at 3000 x rpm at 4°C for 15 minutes. Plasma was added to a cryovial pre-spiked, less than 6 hours prior to plasma collection and stored in a refrigerator set to maintain 4 °C, with 10.0 µL of protease inhibitor cocktail (Sigma, P8340) per 1.0 mL of plasma. Samples were placed on dry ice prior to storage in an ultralow freezer set to maintain -80 °C

#### *Rat Tissue Homogenates*

Weighed tissues were kept on dry ice and minced while still partially frozen. Minced tissues were transferred to a polypropylene tube and combined with Lysing Matrix S balls (MP Biomedical) and Lysis buffer (RIPA buffer (Sigma R0278) supplemented with EDTA free Halt™ protease inhibitor cocktail) and

held on ice for 10 -15 minutes with intermittent vortexing. Samples were homogenized using a GenoGrinder for 3 cycles of 1750 rpm / 30 seconds followed by 5 minutes of cooling on ice followed by centrifugation at 1000*g* at 22°C for 1 minute. Fresh RIPA buffer containing EDTA free Halt™ protease inhibitor cocktail was added to the cell debris and further homogenize by performing 3 cycles of beads beading at 1750 rpm / 30 seconds. The homogenate was centrifuged (1000 g for 5 minutes) and the supernatant combined with the corresponding homogenate from the first round. Homogenates were stored frozen in a -70°C freezer until LC-MS/MS analysis.

*Rat Mitochondrial Extracts*

Mitochondrial extracts were prepared using the commercial kit from Abcam (ab110168) following manufacturer instructions.

*Monkey Tissues (platelets, skin, buccal swabs)*

Venous blood was collected in ACD Vacutainers (Acid Citrate Dextrose (ACD) – Glass Catalog no.364606). Blood (4mL) was transferred to polypropylene tubes and centrifuged at 200*g* for 13 minutes (room temperature, no brakes). The upper platelet-rich plasma layer was then transferred to a new conical tube and spun again at 400*g* for 10 min. The plasma was removed and the platelet pellet was then carefully washed twice with platelet wash buffer (10 mM Sodium Citrate, 150 mM NaCl, 1 mM EDTA, 1% Glucose w/v, pH 7.4 ) by spinning at 800*g* for 5 min after each wash. Protein extraction was completed by addition of 1 mL of RIPA buffer (Sigma R0278) containing EDTA free Halt™ protease inhibitor cocktail to the isolated platelet pellet followed by brief vortexing. Samples were incubated on ice for 10 min with intermittent vortexing. Samples were then sonicated (30 seconds in a sonicator bath) and the platelet lysates were stored frozen in a -70°C freezer until LC-MS/MS analysis.

Skin punch biopsies (8 mm) were partially thawed and minced while kept on dry ice. RIPA buffer (Sigma R0278), containing EDTA free Halt™ protease inhibitor cocktail was added to the minced tissue, incubated on ice for 10 minutes and placed in a sonicator bath for 15 seconds. After sonication the samples were homogenized by bead beating using Lysing Matrix D beads (MP Biomedicals) on the FastPrep-96™ grinder (MP Biomedicals) for 30 seconds at 1800 rpm. The sonication followed by homogenization was repeated twice after which they were centrifuged at 1000 g for 1 minute and the supernatant transferred to a clean tube. Fresh RIPA buffer containing EDTA free Halt™ protease inhibitor cocktail was added to the cell debris and further homogenize by performing 3 cycles of beads beading and sonication. The homogenate was centrifuged (1000 g for 1 minute) and the supernatant combined with the corresponding homogenate from the first round. Homogenates were stored frozen in a -70°C freezer until LC-MS/MS analysis.

Buccal cells (4 swabs per animal, 2 from each of the left and right cheek areas) were collected using Isohelix Swabs SK-2S as per manufacturer's instructions. Swabs were snap frozen and stored frozen until extraction. Protein extraction was completed by addition of RIPA buffer (Sigma R0278) containing EDTA free Halt™ protease inhibitor cocktail to each buccal swab followed by brief vortexing. Samples were incubated on ice for 10 min with intermittent vortexing. After incubation, samples were sonicated (15 seconds in a sonicator bath) followed by centrifugation at 1000 g for 1 minute. The cell lysates were stored frozen in a -70°C freezer until LC-MS/MS analysis.

## Assay quantification methods

### *Nomlabofusp in Mouse Plasma*

Nomlabofusp was measured in mouse plasma using a proprietary electrochemiluminescence sandwich immunoassay and Meso Scale Discovery platform developed at National Center for Advancing Translational Sciences (NCATS). The capture antibody is TAT-specific (anti-TAT) and was generated in rabbits. Rabbit antibodies to the 12aa TAT antigen were raised against KLH-TAT, and affinity purified. Rabbit hybridomas were also selected by screening against BSA-TAT (counterscreened against BSA) and selected clones were verified by biolayer interferometry before scaling up and antibody purification from supernatant by ProteinA-Agarose chromatography. A sulfotagged hFXN-specific (anti-FXN) antibody is used for detection, thus the assay is selective for intact nomlabofusp. The LLOQ for this assay was 90.8 pg/ml in plasma.

### *hFXN in Mouse Tissues and Mitochondrial Extracts*

Human frataxin was measured in mouse RIPA homogenates of heart, lung, skeletal muscle, and liver and in liver mitochondrial extracts with a qualified hybrid LC-MS/MS assay using an anti-FXN antibody capture reagent. Tissue homogenates (25 µL) or mitochondrial extracts (50µL) were transferred to a plate and the following was added: 20µL of 150ng/mL SILAC-nomlabofusp, 400µL of Blocker Casein in PBS (ThermoFisher), 20µL of conjugated magnetic beads suspension (Streptavidin Mag Sepharose, Cytiva), coupled with a biotinylated anti-Frataxin mouse monoclonal antibody [Abcam ab110328]). The sample-bead suspension was incubated for 3 hours (at 22°C, 900 rpm). The beads were then washed twice in 200 µL PBS, followed by resuspension in 100 µL of 100 mM $NH_4HCO_3$:ACN 75:25% v/v and 20µL of 0.1 µg/µL Trypsin Gold (Promega). After 16-20 hours of incubation (at 37°C, 825 rpm), 20µL of 10% HCOOH was added to stop the digestion, and the magnetic beads were removed. LC-MS/MS analysis was performed on the final tryptic peptide solution using an Agilent 1100 HPLC coupled to a SCIEX 6500+ triple quadrupole mass spectrometer, using a column HALO Peptide ES-C18, 50x2.1mm, 2.7µm, with mobile phase 0.1% Acetic Acid in water and in acetonitrile. The "SGT peptide" (a signature peptide with the nomlabofusp/hFXN amino acid sequence SGTLGHPGSLDETTYER, see Schematic below) was monitored (Q1 m/z 607.3; Q3 m/z 669.3). The lower limit of quantitation was 1 ng/g of tissue.

### *Nomlabofusp in Rat Plasma*

Nomlabofusp was measured in rat plasma using a qualified hybrid LC-MS/MS assay that employs an immuno-affinity selection step with an anti-TAT antibody. In brief, 200 µL of rat plasma was incubated with 20 µL SILAC-nomlabofusp (0.5 µg/mL), 20 µL biotinylated anti-TAT antibody (50 µg/mL), and 200 µL of 600 mM acetic acid for 60 minutes at 850 rpm, 22°C. This was followed by the addition of 150 µL of 1M $NH_4HCO_3$ and a second incubation for 60 minutes at 850 rpm, 22°C. The samples were centrifuged for 5 minutes at 2000 x g and mixed with 20 µL of conditioned Dynabeads™ MyOne™ Streptavidin C1 (ThermoFisher). After incubation of the bead/sample mixture for 60-75 minutes at 850 rpm and 22°C, beads were then washed twice in 200 µL PBS, followed by resuspension in 100 µL of 100 mM $NH_4HCO_3$:ACN 75:25% v/v and 20µL of 0.1 µg/µL Trypsin Gold (Promega). After overnight incubation (at 37°C, 850 rpm), 20µL of 10% HCOOH was added to stop the digestion and the magnetic beads were removed. LC-MS/MS was conducted on the final tryptic peptide mixture using UHPLC (Shimadzu Nexera X2; reversed-phase with a Sciex API 6500+ MS/MS) with a column Acquity UPLC Peptide BEH C18 300A 50x 2.10mm, 1.7 uM. The SGT peptide (SGT) (Q1 m/z 607.3; Q3 m/z 669.3) was monitored as a surrogate of nomlabofusp for quantitation, with a calibration range from 0.4 ng/mL to 40 ng/mL. Since the method uses a specific anti-TAT antibody for selection and the monitored SGT peptide belongs to the human frataxin portion of the molecule, the method is selective for intact nomlabofusp (see Schematic below).

*hFXN and Endogenous FXN in Rat Tissues*

Frataxin (human and rat endogenous FXN) were measured in rat tissue homogenates using a qualified hybrid LC-MS/MS assay that employs an immunoaffinity selection step with an anti-frataxin antibody (Abcam, ab113691). This antibody was used to capture rat and human FXN from tissue homogenate samples. Sample homogenates (250 µL) were transferred to a plate and incubated with 20 µL of SILAC-nomlabofusp (150 ng/mL),20 µL of Antibody Conjugated Beads (Streptavidin Mag Sepharose beads, Cytiva), and 200 µL PBS for 2.5 hours (at 22°C and 850-950 rpm). The beads were then washed twice in 500 µL PBS, followed by resuspension in 100 µL of 100 mM $NH_4HCO_3$:ACN 75:25% v/v and 20µL of 0.1 µg/µL Trypsin Gold (Promega). After 16-24 hr incubation (at 37°C, 850 rpm-950 rpm), 20µL of 10% HCOOH was added to stop the digestion, and the magnetic beads were removed. The samples were transferred to a new plate and centrifuged (3828 g, 22°C, 5 min) and stored at 4°C nominal until injection on an UHPLC. LC-MS/MS of the final tryptic peptide mixture was conducted using UHPLC (Shimadzu Nexera X2; reversed-phase with a Sciex API 6500+ MS/MS) with a column Acquity UPLC Peptide BEH C18 300A 50x 2.10mm, 1.7 uM. The trypsin digestion was used to produce two analytes: the SGT peptide (Q1 m/z 607.3; Q3 m/z 669.3) and the LGG peptide (a signature peptide with the amino acid sequence LGGDLGTYVINK (Q1 m/z 625.3; Q3 m/z 794.4)) that is common to both rat and human FXN (see Schematic below). The calibration ranged from 0.25 ng/mL to 50 ng/mL. The SGT peptide (human FXN specific tryptic peptide) was monitored as a surrogate of hFXN and the LGG peptide (pan-species FXN specific tryptic peptide) was monitored as a surrogate of total FXN (rat and human) from tissue homogenates using LC-MS/MS. The endogenous rat FXN level from a post-treatment sample was obtained by subtracting the hFXN (SGT) level from the pan-specific peptide (LGG) level, i.e. LGG-SGT. A bicinchoninic acid (BCA) protein quantification method (ThermoFisher) was also used to quantify total protein in the tissue homogenates so that the final concentration of frataxin was normalized to the total protein content. The total protein was quantified over a theoretical concentration range of 50.0 µg/mL to 2000.0 µg/mL.

*Nomlabofusp, hFXN and Endogenous FXN in Monkey Platelets, Buccal and Skin*

Frataxin (human and cynomolgus monkey endogenous FXN) were measured in monkey tissue homogenates using a hybrid LC-MS/MS assay that employs an immuno-affinity selection step with an anti-frataxin antibody (Abcam ab110328). This antibody was used to capture nomlabofusp, human FXN and NHP (cynomolgus monkey) FXN from tissue homogenate samples. Sample homogenates (250 µL) were transferred to a plate and incubated with 20 µL of SILAC (150 ng/mL), 20 µL of Antibody Conjugated Beads (Streptavidin Mag Sepharose, Cytiva), and 250 µL Blocker™ Casein in PBS (ThermoFisher) for 3 hours (at 22°C and 900 rpm). The beads were washed twice in 500 µL PBS, followed by resuspension in 100 µL of 100 mM $NH_4HCO_3$:ACN 75:25% v/v and 20µL of 0.1 µg/µL Trypsin Gold (Promega). After 16-20 hr incubation (at 37°C, 825 rpm), 20µL of 10% HCOOH was added to stop the digestion, and the magnetic beads were removed. The samples were transferred to a new plate and stored at 4°C nominal until injection on an UHPLC. LC-MS/MS analysis was performed on the final tryptic peptide solution using an Agilent 1100 HPLC coupled to a SCIEX 6500+ triple quadrupole mass spectrometer, using a column HALO Peptide ES-C18, 50x2.1mm, 2.7µm, with mobile phase 0.1% Acetic Acid in water and in acetonitrile. Due to the species homology between human and monkey FXN, three analytes were used to distinguish nomlabofusp from human and monkey FXN (see Schematic below): 1) the SGT peptide (Q1 m/z 607.3; Q3 m/z 669.3) which is a human FXN peptide that is also present in nomlabofusp, 2)the LGG peptide (Q1 m/z 625.3; Q3 m/z 794.4) that corresponds with both human and monkey FXN, and 3) the GGM peptide (a signature peptide with the amino acid sequence GGMWTLGR (Q1 m/z 439.2; Q3 m/z 632.3)) that is located at the junction of the TAT and the MTS, which is specific for nomlabofusp. The calibration ranged from 0.25 ng/mL to 50 ng/mL. A BCA protein quantification

method was also used to quantify total protein concentrations of the tissue homogenates so that the final concentration of the LC-MS/MS analytes were normalized to the total protein levels. The total protein was quantified over a theoretical concentration range of 50.0 µg/mL to 2000.0 µg/mL using a commercial BCA assay kit (ThermoFisher).

*Nomlabofusp and FXN in Monkey CSF*

Frataxin was quantified in CSF samples utilizing an Enzyme Linked Immunosorbant Assay (ELISA) kit (Abcam, ab176112) for 1 x 96 well assay plate, as per manufacturer instructions. Reference standard calibration curves were prepared in assay buffer and 10% CSF and the range of quantitation was 9.88 to 267 pg/mL. All CSF samples were pre-diluted in assay buffer at 1:10, 1:100 and 1:1000. The antibody used in this assay cross-reacts with hFXN and nomlabofusp, hFXN is given by subtracting the nomlabofusp concentration.

Nomlabofusp was quantified by ELISA method using a High Bind ELISA plate with a proprietary anti-TAT affinity purified rabbit polyclonal antibody. An anti-frataxin detection antibody (Abcam ab176112) conjugated to horseradish peroxidase is used to detect the bound CTI-1601 on the plate. The color development was stopped with sulfuric acid and the spectrophotometric plate reader captures absorbance at 450nm and 650nm. The absorbance at 650nm is subtracted from the reading at 450 nm. The minimum required dilution for this assay is an MRD of 10. The range of quantitation for the analysis of these CSF samples extended from 0.800 to 25.6 ng/mL.

***Schematic representation of nomlabofusp, and the hybrid LC-MS/MS methods used for nomlabofusp or FXN measurement.*** *Nomlabofusp is a recombinant fusion protein consisting of a transactivator of transcription (TAT) cell penetrant peptide (maroon) fused via a diglycine linker (yellow) to the amino terminus of the full-length human frataxin (FXN) molecule comprising the native mitochondrial targeting sequence (MTS) (green) and mature FXN (blue). Nomlabofusp and FXN are quantified using separate but similar hybrid LC-MS/MS methods, based on specific antibody-mediated immunocapture followed by the monitoring of unique signature peptides, characterized in the associated table below the illustration.*

**Hybrid LC-MS/MS Assay Formats**

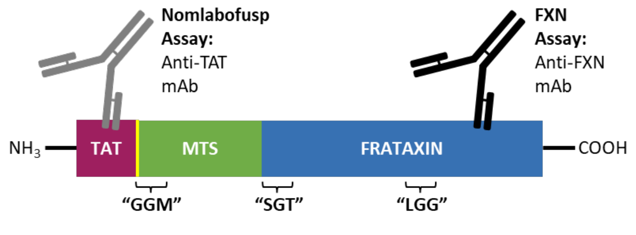


| TRYPTIC PEPTIDE | CHARACTERISTICS |
|---|---|
| **GGM:** GGMWTLGR | • Specific for nomlabofusp<br>• Absent in mature human FXN |
| **SGT:** SGTLGHPGSLDETTYER | • Specific to nomlabofusp, immature human FXN and mature human FXN<br>• Absent in mouse, rat and monkey proteomes and thus does not detect FXN in these species |
| **LGG:** LGGDLGTYVINK | • Specific to nomlabofusp, immature human FXN and mature human FXN<br>• Present in mouse, rat and monkey proteomes and thus detects FXN in these species as well |
