# Supplementary material for: Pharmacokinetics and Pharmacodynamics of Nomlabofusp in Non-clinical Studies of Friedreich’s Ataxia
Source: AAPS J. Author manuscript; Available in PMC 2026 May 5. (PMC13143400; doi:10.1208/s12248-025-01093-y)
Supplement: Suppl 1 [file NIHMS2151153-supplement-Suppl_1.pdf]

# SUPPLEMENTARY FILE 1

**Anesthetized Echocardiography was performed by the JAX In Vivo Imaging and Physiology (IVIP) Core Facility (Jackson Laboratories, Bar Harbor, ME) as follows:**

Echocardiac assessments were carried out using the Vevo 770/2100 high frequency ultrasound with 30 and 40MHz probes (VisualSonics Inc. Toronto, Canada). Mice were anesthetized with up to 5% isoflurane at a flow rate of 0.8-2.0L/min in oxygen. Echocardiography used pulsed Doppler sonography applied through the ultrasound probe to measure blood flow rates, volumes and cardiac dynamics. All testing was carried out using Power Lab acquisition hardware with LabChart Analysis Software (AD Instruments, Norwood, MA). The following definitions and calculations for echocardiography were applied: Heart Rate (HR [BPM]), Average 5 beats; Left Ventricle Stroke Volume (LV SV [µl]), V; d - V; s; Left Ventricle Ejection Fraction (EF [%]) , (V; d - V; s) / V; d x 100; Left Ventricle Fractional Shortening (FS [%]), (D ; d -D s) / D; d x 100; Left Ventricular Cardiac Output (LV CO [mL/min]), Heart Rate x Stroke Volume; Left Ventricle Internal Dimension (D;s [mm]); Left Ventricle Volume, systole (V; s [µl]), [7.0 / (2.4 + D; s)] x (D; s)3.
